# Bortezomib-induced miRNAs direct epigenetic silencing of locus genes and trigger apoptosis in leukemia

**DOI:** 10.1038/cddis.2017.520

**Published:** 2017-11-09

**Authors:** Yu-Yi Chu, Chiung-Yuan Ko, Shao-Ming Wang, Pin-I Lin, Han-Ying Wang, Wen-Chi Lin, Dong-Yu Wu, Lu-Hao Wang, Ju-Ming Wang

**Affiliations:** 1 Institute of Bioinformatics and Biosignal Transduction, National Cheng Kung University, Tainan, Taiwan; 2 Department of Biotechnology and Bioindustry Sciences, College of Bioscience and Biotechnology, National Cheng Kung University, Tainan, Taiwan; 3 The Ph.D. Program for Neural Regenerative Medicine, Taipei Medical University, Taipei, Taiwan; 4 Center for Neurotrauma and Neuroregeneration, College of Medical Science and Technology, Taipei Medical University, Taipei, Taiwan; 5 Institute of Basic Medical Sciences, National Cheng Kung University, Tainan, Taiwan; 6 Graduate Institute of Medical Sciences, College of Medicine, Taipei Medical University, Taipei, Taiwan

## Abstract

MicroRNAs (miRNAs) have been suggested to repress transcription via binding the 3′-untranslated regions of mRNAs. However, the involvement and details of miRNA-mediated epigenetic regulation, particularly in targeting genomic DNA and mediating epigenetic regulation, remain largely uninvestigated. In the present study, transcription factor CCAAT/enhancer binding protein delta (CEBPD) was responsive to the anticancer drug bortezomib, a clinical and highly selective drug for leukemia treatment, and contributed to bortezomib-induced cell death. Interestingly, following the identification of CEBPD-induced miRNAs, we found that miR-744, miR-3154 and miR-3162 could target CpG islands in the 5′-flanking region of the *CEBPD* gene. We previously demonstrated that the Yin Yang 1 (YY1)/polycomb group (PcG) protein/DNA methyltransferase (DNMT) complex is important for CCAAT/enhancer binding protein delta (CEBPD) gene inactivation; we further found that Argonaute 2 (Ago2) interacts with YY1 and binds to the *CEBPD* promoter. The miRNA/Ago2/YY1/PcG group protein/DNMT complex linked the inactivation of *CEBPD* and genes adjacent to its 5′-flanking region, including protein kinase DNA-activated catalytic polypeptide (*PRKDC*), minichromosome maintenance-deficient 4 (*MCM4*) and ubiquitin-conjugating enzyme E2 variant 2 (*UBE2V2*), upon bortezomib treatment. Moreover, we revealed that miRNA binding is necessary for YY1/PcG group protein/DNMT complex-mediated epigenetic gene silencing and is associated with bortezomib-induced methylation on genomic DNA. The present study successfully characterized the interactions of the miRNA/Ago2/YY1/PcG group protein/DNMT complex and provided new insights for miRNA-mediated epigenetic regulation in bortezomib-induced leukemic cell arrest and cell death.

MicroRNAs (miRNAs) are composed of 21–24 RNA nucleotides that regulate multiple genes and cellular functions in tumor progression.^1, 2^ By interacting with members of the Argonaute (Ago) protein subfamily, miRNAs primarily target homologous sites in the untranslated regions (UTRs) of mRNAs, thereby contributing to translational inhibition or mRNA degradation.^3, 4^ Based on the findings of transcriptome analysis, over 70% of gene promoters overlapped with noncoding RNA transcripts, which might serve as miRNAs target sites.^5, 6^ Although miRNAs primarily mediate post-transcriptional gene silencing (PTGS) in the cytoplasm, recent studies have shown that miRNAs also translocate to the nucleus and interact with promoter DNA, thus inactivating gene expression.^7^ In addition, exogenous small RNAs have been implicated in Ago-mediated histone modification and DNA methylation of gene transcripts^8, 9, 10^ and are known for transcriptional gene silencing (TGS). Argonaute 2 (Ago2) has the most well-established role in RNAi as the catalytic engine that drives mRNA cleavage.^11^ Ago1 and Ago2 have positively charged surfaces that are well suited for binding RNA and aligning it with a complementary nucleic acid. Previous studies have shown that Importin 8 mediates the nuclear transport of miRNAs and the associated Ago proteins, which regulate chromatin modification.^12, 13^ However, the consequent effects and mechanisms in response to the miRNA-mediated epigenetic regulation remain largely uninvestigated.

Transcription factors have been suggested to regulate the biogenesis of miRNAs.^14, 15^ CCAAT/enhancer binding protein delta (CEBPD) is a transcription factor that contributes to cell differentiation, motility and death.^16, 17^ The abundance of CEBPD is typically low in most cells at normal physiological conditions, but is rapidly induced through external stimuli.^18, 19^ However, the biology of miRNAs in response to CEBPD activation and consequent effects remain largely unknown. We previously demonstrated that CEBPD could serve as a tumor suppressor through the activation of apoptosis and growth arrest in prostate, cervical and breast cancers.^18, 20, 21, 22^ We have also demonstrated that CEBPD expression can be attenuated in cancer cells by Yin Yang 1 (YY1)/polycomb group (PcG)/DNA methyltransferase (DNMT)-mediated epigenetic regulation.^20, 22^ In leukemia, CEBPD was suppressed in the blast crisis phase of chronic myeloid leukemia^23^ and acute myeloid leukemia (AML) patients.^24^ However, the CEBPD-mediated and detailed regulation, especially in response to anticancer drugs, in leukemia is still an open question.

Bortezomib, the first selective inhibitor of the proteasome to reach clinical trials, causes G2–M cell cycle arrest and apoptosis by blocking the action of the 26S proteasome.^25^ Bortezomib has been shown to have *in vitro* and *in vivo* activity against a variety of malignancies, including multiple myeloma,^26^ chronic lymphocytic leukemia,^27^ prostate cancer,^28^ pancreatic cancer^29^ and colon cancer.^30^ A recent study showed that bortezomib enhances the efficacy of volasertib-induced mitotic arrest in AML *in vitro* and prolongs survival *in vivo*.^31^ Although the transcription-associated apoptotic activation in response to bortezomib has been suggested,^32, 33^ the mechanisms and new insights, especially in PTGS-mediated regulation, remain largely uninvestigated.

In this study, CEBPD-responsive miRNAs were identified in leukemic cells. Moreover, protein kinase DNA-activated catalytic polypeptide (*PRKDC*), minichromosome maintenance deficient 4 (*MCM4*) and ubiquitin-conjugating enzyme E2 variant 2 (*UBE2V2*) genes, which are located in the 5′ upstream region of the *CEBPD* gene, are important for DNA repair and cell cycle regulation.^34, 35, 36^ We found that CEBPD-responsive miR-744, miR-3154 and miR-3162 could bind to the promoter regions of CEBPD and its 5′ upstream genes *PRKDC*, *MCM4* and *UBE2V2*. In certain types of cancer, CEBPD activity is silenced by the epigenetic regulators YY1, PcG complex and DNMT via enhanced DNA methylation.^20, 37^ Moreover, we further demonstrated that the miRNAs, in response to CEBPD, could interact with Ago2 and contribute to histone and DNA methylation via recruiting the binding of YY1/PcG protein/DNMT complex. This binding consequently inhibited CEBPD and its 5′ upstream genes *PRKDC*, *MCM4* and *UBE2V2*. The novel miRNA/Ago2-mediated regulation provided a new insight into bortezomib-induced cell death in leukemic cells.

## Results

### CEBPD is activated by bortezomib and contributes to bortezomib-induced leukemic cell apoptosis

**Figure 1:**
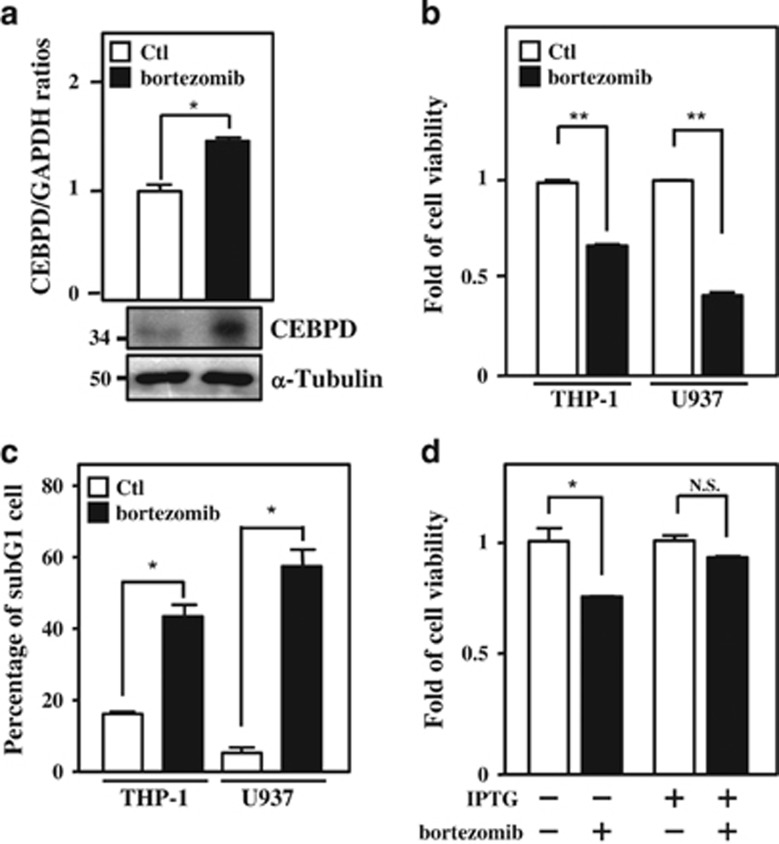
CEBPD, which contributes to leukemic cell apoptosis, is responsive to bortezomib. (**a**) Bortezomib increases the expression of CEBPD. After treatment with bortezomib (50 nM) for 6 h in THP-1 cells, total RNA and protein lysates were harvested and analyzed using qPCR and western blot analysis. (**b**) Bortezomib inhibits cell viability. After treatment with bortezomib (50 nM) for 24 h, the viability of leukemic cells (THP-1 and U937) was measured using an MTT assay. (**c**) Bortezomib induces the growth arrest of leukemic cells. After treating with bortezomib (50 nM) for 24 h, the sub-G1 phase of THP-1 and U937 cells was analyzed by flow cytometry. (**d**) Attenuated CEBPD reverses bortezomib-reduced cell viability in THP-1 cells. THP-1 cells with the IPTG-inducible CEBPD knockdown system were pretreated with IPTG (500 *μ*M) for 3 h. After treatment with bortezomib (50 nM) for 24 h, cell viability was measured using an MTT assay. The data are presented as the mean±standard error of experiments performed in triplicate (*P<0.05, **P<0.01, Student’s *t*-test). NS, not significant

**Figure 2:**
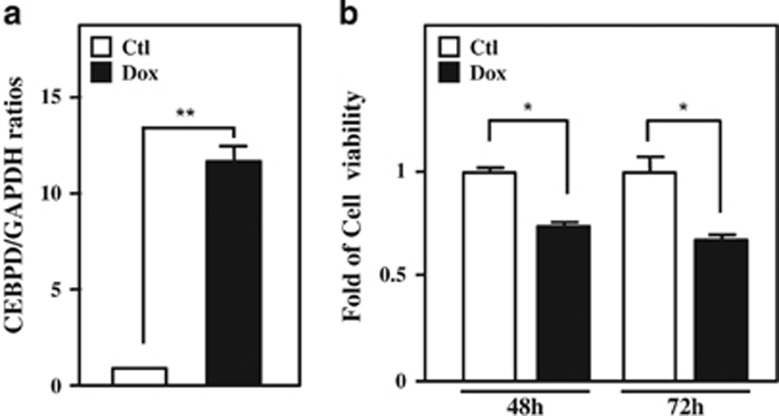
CEBPD inhibits the survival rates of THP-1 cells. (**a**) Dox successfully induces CEBPD expression in THP-1 cells in the Dox-inducible CEBPD expression system. THP-1 cells were treated with Dox (1 *μ*g/ml) for 6 h, followed by qPCR to analyze the expression of CEBPD. (**b**) CEBPD reduces the viability of THP-1 cells. THP-1 cells with the Dox-inducible CEBPD expression system were treated with Dox (1 *μ*g/ml), and cell viability was measured using an MTT assay. The data are presented as the mean±standard error of experiments performed in triplicate (*P<0.05, **P<0.01, Student’s *t*-test). NS, not significant

CEBPD was suggested to respond to many anticancer drugs and to contribute to apoptosis induced by these anticancer drugs.^38, 39^ The anticancer drug bortezomib was originally suggested to serve as a proteasome inhibitor and a common therapeutic drug of leukemia. Following the observation that CEBPD was responsive to bortezomib treatment in leukemic cells (Figure 1a and Supplementary Figure S1a), we further examined the effect of bortezomib in the induction of apoptosis and tested the involvement of CEBPD in the bortezomib-induced death of leukemic cells. 3-(4,5-dimethylthiazol-2-yl)-2,5-diphenyl tetrazolium bromide (MTT) assay and flow cytometry were performed to examine cell survival and assess the sub-G1 cell population, respectively. Reduced cell viability and increased sub-G1 population and apoptosis of THP-1 and U937 cells were observed upon bortezomib treatment (Figures 1b and c and Supplementary Figure S2). Furthermore, an attenuated apoptotic effect was observed following the depletion of CEBPD in bortezomib-treated THP-1 cells (Figure 1d). Next, we verified the involvement of CEBPD in the induction of apoptosis of leukemic cells. A cell viability assay was performed via a doxycycline (Dox)-inducible CEBPD expression system to verify the proapoptotic role of CEBPD in leukemic cells. As expected, induction of CEBPD could attenuate the viability of THP-1 cells (Figures 2a and b). These data suggested that CEBPD has a proapoptotic role in bortezomib-induced apoptosis of leukemic cells.

### CEBPD negatively autoregulates and coordinately inactivates the genes located in the 5′ upstream region of the *CEBPD* gene

**Figure 3:**
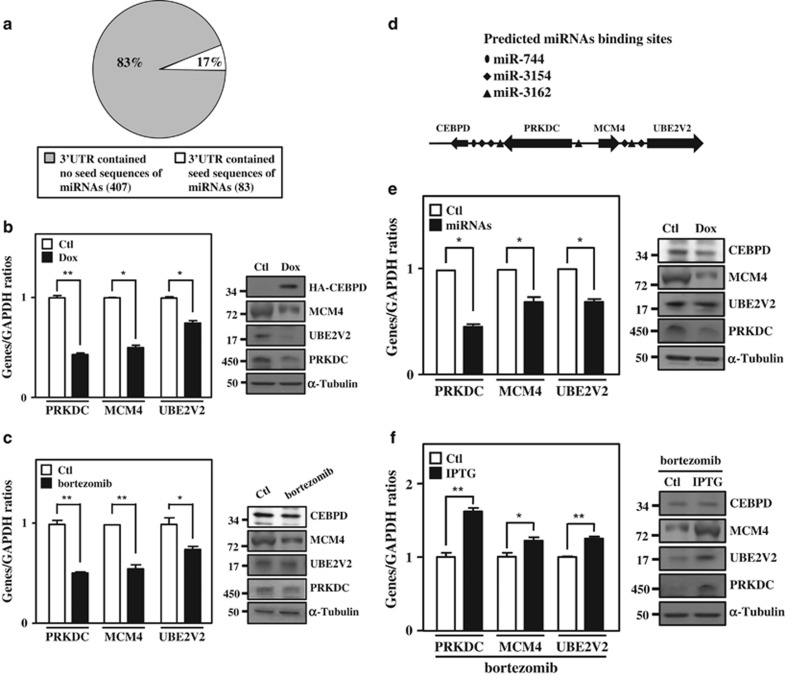
CEBPD and responsive miRNAs contribute to the inactivation of the surrounding genes. (**a**) The 3′-UTRs of most CEBPD-downregulated mRNAs are not targeted by CEBPD-upregulated miRNAs. The CEBPD-upregulated miRNAs and -downregulated mRNAs microarray profiling was analyzed using the miRTar prediction program. The pie chart presents the percentage of CEBPD-downregulated mRNAs in the 3′-UTR, containing the seed sequences of CEBPD-upregulated miRNAs. (**b**) CEBPD participates in the inactivation of the surrounding genes. THP-1 cells with the Dox-inducible CEBPD expression system were treated with Dox (1 *μ*g/ml) for 18 h; total RNA was harvested to perform qPCR (left panel), and total protein was harvested to perform western blot analysis (right panel). (**c**) Bortezomib represses the expression of *PRKDC*, *MCM4* and *UBE2V2*. After treating with bortezomib (50 nM) for 16 h, total RNA was harvested to perform qPCR (left panel) and total protein was harvested to perform the western blot analysis (right panel). (**d**) Schematic representation showing the location of genes and putative miRNA-binding motifs. (**e**) miR-744, miR-3154 and miR-3162 reduces CEBPD surrounding genes. THP-1 cells with the Dox-inducible miR-744, miR-3154 and miR-3162 expression system were treated with Dox (1 *μ*g/ml) for 18 h. Total RNA was harvested to perform qPCR (left panel), and total protein was harvested to perform the western blot analysis (right panel). (**f**) Attenuated CEBPD reverses bortezomib-inhibited gene expression in THP-1 cells. THP-1 cells with the IPTG-inducible CEBPD knockdown system were pretreated with IPTG (500 *μ*M) for 3 h. After treatment with bortezomib (50 nM) for 16 h, total RNA was harvested to perform qPCR (left panel) and total protein was harvested to perform the western blot analysis (right panel). The data are presented as the mean±standard error of experiments performed in triplicate (*P<0.05, **P<0.01, Student’s *t*-test)

CEBPD is a well-known transcription factor and has been suggested to promote apoptosis through the activation of apoptotic genes.^21, 22^ However, the involvement of CEBPD in miRNA regulation and its association with consequent cellular responses, especially in its proapoptotic role, remain poorly understood. We first performed miRNA and mRNA microarray analysis in THP-1 cells to explore the link between CEBPD-regulated miRNAs and mRNAs. Using the public access program miRTar, we explored the correlation between the CEBPD-induced downregulated mRNA (Supplementary Table 1) and upregulated miRNA profiles (Supplementary Table 2). Interestingly, among 490 CEBPD-downregulated mRNAs, 407 (83%) CEBPD-responsive mRNAs were not targeted by CEBPD-responsive miRNAs in their UTRs (Figure 3a and Supplementary Table 3). These findings implied that downregulated mRNAs that respond to CEBPD activation could be regulated via other UTR-independent mechanisms. Recently, miRNAs were suggested to be involved in epigenetic regulation via directly targeting DNA.^40, 41^ We found that several top 10-ranked CEBPD-responsive miRNAs showed a binding potential on the 5′-flanking region of the *CEBPD* gene. As mentioned above, the *PRKDC*, *MCM4* and *UBE2V2* genes, which are located in the 5′ upstream region of the *CEBPD* gene, have a potent role in promoting cell cycle S-phase entry or inhibiting apoptosis.^34, 35, 36^ Importantly, we found that exogenously expressing CEBPD could attenuate endogenous *CEBPD* transcripts (Supplementary Figure S3). Moreover, although the transcription of the *PRKDC*, *MCM4* and *UBE2V2* genes was not immediately responsive to bortezomib treatment, their transcription was coordinately attenuated with the inactivation of CEBPD in long-term bortezomib treatment (Supplementary Figure S4). These observations raised our interests to test whether activated CEBPD could upregulate miRNAs to inactivate the transcription of *CEBPD* itself, and the *PRKDC*, *MCM4* and *UBE2V2* genes in bortezomib-treated leukemic cells.

Quantitative PCR results showed that *PRKDC*, *MCM4* and *UBE2V2* transcripts were attenuated following the induction of CEBPD after exogenously expressing CEBPD or bortezomib treatment in THP-1 or U937 cells (Figures 3b and c and Supplementary Figure S1b). Meanwhile, by analysis with miRTar program, no putative CEBPD-responsive miRNA seed sequences were predicted in the UTRs of *CEBPD*, *PRKDC*, *MCM4* and *UBE2V2* mRNAs. Surprisingly, the complementary seed sequences of CEBPD-responsive miR-744, miR-3154 and miR-3162 (Supplementary Table 2) were predicted to bind the promoter regions of *PRKDC*, *MCM4* and *UBE2V2* genes (Figure 3d). We therefore tested whether *PRKDC*, *MCM4* and *UBE2V2* mRNAs could be regulated by these three miRNAs. The results showed that *PRKDC*, *MCM4* and *UBE2V2* transcripts were downregulated following the increase of these three miRNAs (Figure 3e and Supplementary Figure S8a). Interestingly, in addition to these three genes, the exogenous expression of CEBPD also attenuated endogenous *CEBPD* transcripts (Supplementary Figure S3a). The result implied that induction of CEBPD could negatively autoregulate *CEBPD* transcription in leukemic cells. Furthermore, the loss of CEBPD increased the expression of *PRKDC*, *MCM4* and *UBE2V2* upon bortezomib treatment (Figure 3f). The results raised a speculation that *CEBPD* could be the first responsive gene following bortezomib treatment; moreover, the induction of CEBPD increased the expression of miR-744, miR-3154 and miR-3162. Subsequently, these miRNAs could feedback to suppress the transcription of *CEBPD* itself and *PRKDC*, *MCM4* and *UBE2V2* genes.

### CEBPD activates the transcription of miR-744, miR-3154 and miR-3162

**Figure 4:**
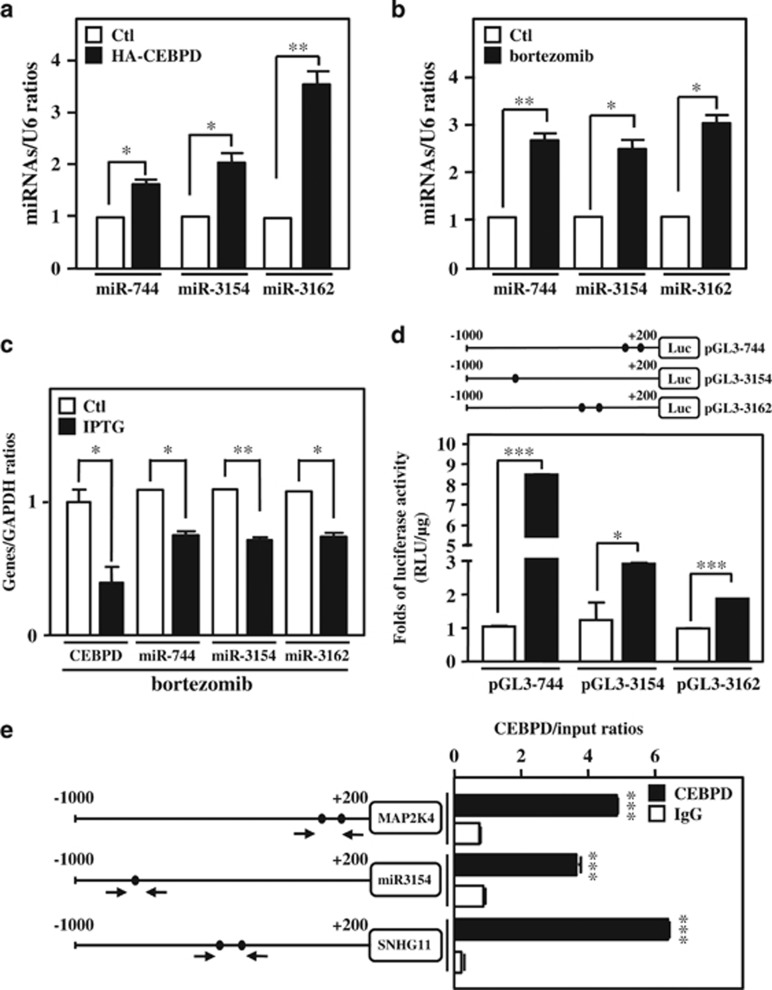
miR-744, miR-3154 and miR-3162 are CEBPD-responsive miRNAs. (**a**) CEBPD induces miR-744, miR-3154 and miR-3162 expression. THP-1 cells were transfected with HA-CEBPD for 18 h. Total RNA was harvested, and the levels of miRNAs (miR-744, miR-3154 and miR-3162) were confirmed by qPCR. (**b**) Bortezomib increases miR-744, miR-3154 and miR-3162 expression. qPCR was performed using total RNA harvested from THP-1 cells treated with bortezomib (50 nM) for 6 h. (**c**) The loss of CEBPD reduced bortezomib-induced miR-744, miR-3154 and miR-3162. THP-1 cells with IPTG-inducible CEBPD knockdown system were pretreated with IPTG (500 *μ*M) for 3 h. After treatment with bortezomib (50 nM) for 6 h, the expression levels of miR-744, miR-3154 and miR-3162 were measured using qPCR. (**d**) CEBPD activates the promoter activity of miRNAs. Schematic representation of the reporter constructs with the miR-744, miR-3154 and miR-3162 promoters. The approximate location of putative CEBPD-binding motifs is indicated with an oval. The luciferase activity was assessed after co-transfecting reporters and expression vectors in THP-1 cells as indicated. (**e**) CEBPD directly binds to the miR-744, miR-3154 and miR-3162 promoters *in vivo*. A ChIP assay was performed in THP-1 cells with the Dox-inducible CEBPD expression system. Sonicated chromatin was subjected to ChIP-qPCR analysis using CEBPD or control IgG antibodies. The data are presented as the mean±standard error of experiments performed in triplicate (*P<0.05, **P<0.01, ***P<0.001, Student’s *t*-test)

To reveal the findings described above, we first examined whether CEBPD could induce miR-744, miR-3154 and miR-3162 transcription by directly activating their promoters. The results showed that exogenous HA/CEBPD and bortezomib treatment could activate miR-744, miR-3154 and miR-3162 transcription in THP-1 and U937 cells (Figures 4a and b and Supplementary Figure S5). Moreover, inactivation of CEBPD attenuated miR-744, miR-3154 and miR-3162 transcripts upon bortezomib stimulation (Figure 4c). In addition, miR-3154 is an intergenic miRNA that has its own promoter. Intragenic miR-744 and miR-3162 belong to two individual genes, *MAP2K4* and *SNHG11*, and overexpressed CEBPD could activate *MAP2K4* and *SNHG11* expression (Supplementary Figure S7). We next investigated whether CEBPD could directly activate the promoters of the genes encoding miR-744, miR-3154 and miR-3162. We cloned and generated three reporters bearing the promoter regions of these three miRNA locations (Figure 4d, upper panel). The results of the reporter assay showed that exogenously expressing CEBPD could induce the promoter activity of genes encoding the three individual miRNAs (Figure 4d, lower panel). Furthermore, an *in vivo* DNA binding assay showed that CEBPD could directly bind to the promoter regions of genes encoding miR-744, miR-3154 and miR-3162 (Figure 4e). These results suggested that CEBPD was responsive to bortezomib and directly activated the transcription of miR-744, miR-3154 and miR-3162.

### Ago2 interacts with YY1 and binds to promoter regions with the PcG complex and HP-1

**Figure 5:**
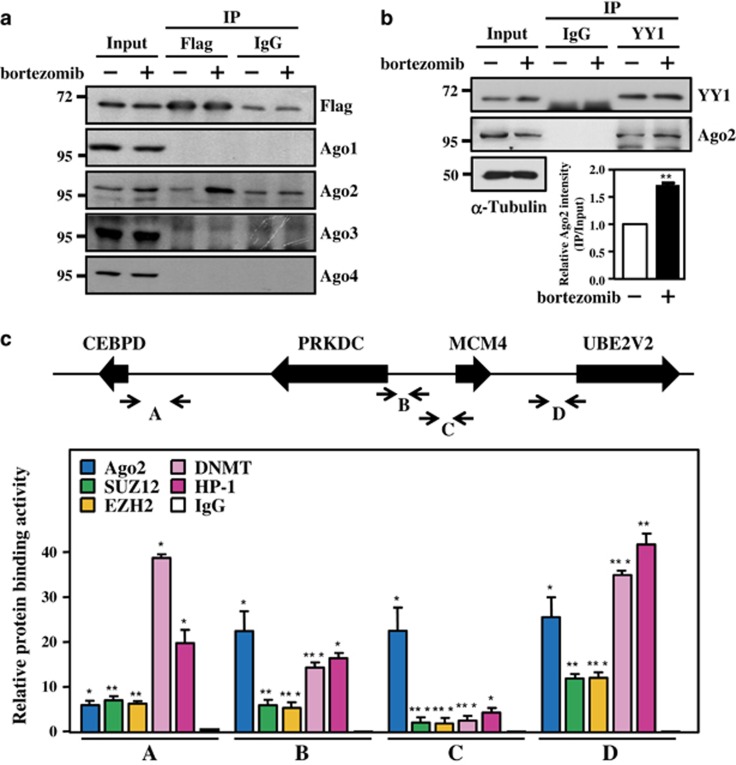
Bortezomib induces the formation of the Ago2/YY1 complex and the binding of the PcG complex. (**a** and **b**) Bortezomib induces Ago2 and YY1 interaction. The 293T cells were transfected with Flag-YY1 and treated with bortezomib (50 nM) for 5 h. Total lysates were collected and analyzed using immunoprecipitation using an anti-Flag antibody (**a**). The THP-1 cells were treated with bortezomib (50 nM) for 12 h. Total lysates were collected and analyzed using immunoprecipitation using an anti-YY1 antibody (**b**, upper panel), and quantitated results showed the interaction intensity between YY1 and Ago2 (**b**, bottom panel). (**c**) The Ago2-PcG complex (SUZ12, DNMT, EZH2) and HP-1 directly bind to the upstream region of CEBPD and the surrounding genes. A ChIP assay was performed using THP-1 cells treated with bortezomib (50 nM). The sonicated chromatin of THP-1 cells was separately immunoprecipitated using specific antibodies against Ago2, SUZ12, DNMT, EZH2, HP-1 and control IgG. The immunoprecipitated DNA was qPCR amplified using different primers as indicated in the top panel. The data are presented as the mean±standard error of experiments performed in triplicate (*P<0.05, **P<0.01, ***P<0.001, Student’s *t*-test)

Mature miRNAs guide Ago-containing complexes to target partially complementary seed sequences on mRNAs and induce the repression of gene expression at the level of mRNA stability or translation. CEBPD activates the expression of miR-744, miR-3154 and miR-3162 in THP-1 cells. Moreover, our current results showed that the increase of these miRNAs could inactivate the transcription of *CEBPD* itself and *PRKDC*, *MCM4* and *UBE2V2* genes. Thus, we next dissected the potent mechanism underlying miRNA-mediated transcriptional silencing on genomic DNA (gDNA). The results of the immunoprecipitation assay showed that Ago2, but not Ago1, could specifically associate with YY1 in 293T and THP-1 cells with or without bortezomib treatment (Figures 5a and b). In addition, our previous study showed that the YY1/PcG/DNMT complex can be recruited to CpG islands in the promoter region of the *CEBPD* gene, and that it is critical for mediating epigenetic silencing of the *CEBPD* gene.^20^ Moreover, the putative YY1-binding motifs could also be predicted in the promoter regions of the *PRKDC*, *MCM4* and *UBE2V2* genes. Thus, we verified whether Ago2, YY1, the PcG complex and DNMT1 can be recruited to the promoter regions of the *CEBPD*, *PRKDC*, *MCM4* and *UBE2V2* genes using a chromatin immunoprecipitation (ChIP)-quantitative PCR (qPCR) assay. The elevated binding of Ago2, YY1, SUZ12, EZH2 and DNMT1 was observed in the regions of the corresponding primers for the indicated promoters upon bortezomib treatment (Figure 5c). In addition, the binding of HP-1, leading to heterochromatin formation and inactivation of genes, was also detectable on the promoter regions of *CEBPD*, *PRKDC*, *MCM4* and *UBE2V2* genes (Figure 5c). These results suggested that Ago2 interacts with YY1 and YY1-interacting proteins, including PcG proteins, DNMT1 and HP1, upon bortezomib treatment.

### miR-744, miR-3154 and miR-3162 mediate TGF by directly binding gDNA

**Figure 6:**
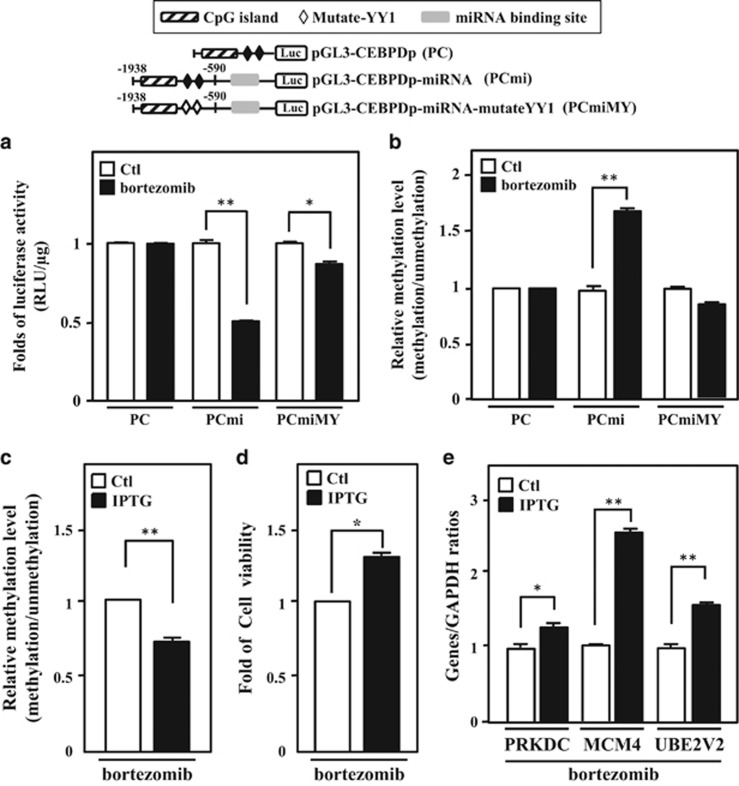
miR-744, miR-3154 and miR-3162 mediates a TGS through a direct binding on gDNA. (**a**) The binding of miRNAs and YY1 is associated with bortezomib-induced epigenetic gene silencing and methylation level. miR-744, miR-3154 and miR-3162 are necessary for YY1/PcG complex-mediated epigenetic gene silencing. Schematic representation of reporter constructs with the *CEBPD* promoter only (PC), *CEBPD* promoter constructed with miRNAs binding sites (PCmi) and PCmi with mutated YY1 binding sites (PCmiMY). The luciferase activity was assessed after transfecting various constructs, as indicated, in 293T cells and subsequently treating the cells with bortezomib (50 nM). (**b**) The binding of miRNAs and YY1 increase the methylation levels of the CpG islands on *CEBPD* promoters. After transfecting constructs into 293T with or without bortezomib (50 nM) treatment, the methylation level of CpG islands on the reporters (PC, PCmi and PCmiMY) was obtained using quantitative methylation-specific PCR. (**c**) miR-744, miR-3154 and miR-3162 are critical for bortezomib-induced DNA methylation. After transfecting constructs into 293T with the IPTG-inducible miR-744/3154/3162-silencing system, the cells were pretreated with IPTG (500 *μ*M) for 3 h and treated with bortezomib (50 nM) for 16 h. Methylation level of CpG islands on the reporter (PCmi) was obtained using quantitative methylation-specific PCR. (**d**) Inhibition of miR-744, miR-3154 and miR-3162 reverses bortezomib-reduced cell viability in THP-1 cells. THP-1 cells with the IPTG-inducible miRNA-744/3154/3162-silencing system were pretreated with IPTG (500 *μ*M) for 3 h. After treating with bortezomib (50 nM) for 24 h, cell viability was measured by the MTT assay. (**e**) Attenuated miR-744, miR-3154 and miR-3162 reverses bortezomib-reduced *PRKDC*, *MCM4* and *UBE2V2* expression. THP-1 cells with the IPTG-inducible miR-744/3154/3162-silencing system were pretreated with IPTG (500 *μ*M) for 3 h. After treating with bortezomib (50 nM) for 16 h, the expression levels of *PRKDC*, *MCM4* and *UBE2V2* were measured by qPCR. The data are presented as the mean±standard error of experiments performed in triplicate (*P<0.05, **P<0.01, Student’s *t*-test)

The partial complementary seed sequences of miR-744, miR-3154 and miR-3162, and the consensus YY1-binding motifs were observed in the promoter region of the *CEBPD* gene. To dissect the binding scenario of miRNAs and YY1 in epigenetic-mediated gene silencing, several heterologous *CEBPD* reporters were constructed (Figure 6, upper panel) for evaluating their individual contribution and regulation. The results of the reporter assay showed that bortezomib had no effect on the pGL3-promoter-CEBPD (PC) reporter (1938/−590 *CEBPD* promoter containing YY1-binding motifs), but significantly repressed the activity of the PC with miRNA binding sites (PCmi) reporter (1938/−590 *CEBPD* promoter region containing YY1-binding motifs and fused with complementary seed sequences of miR-744, miR-3154 and miR-3162). Importantly, the activity of the PCmi with mutated YY1 binding sites (PCmiMY) reporter (−1938/−590 *CEBPD* promoter region containing mutant YY1-binding motifs and fused with complementary seed sequences of miR-744, miR-3154 and miR-3162) was reversed upon bortezomib treatment (Figure 6a). In addition, our previous study demonstrated that YY1 could recruit DNMTs and methylate the CpG islands on the *CEBPD* promoter.^20^ Next, to investigate whether miRNAs could affect the DNA methylation status, we recovered the transfected reporters and treated samples with bisulfite, followed by methylation-specific PCR. We found that bortezomib enhanced the DNA methylation in the −1938/−590 *CEBPD* promoter region of PCmi, but that the methylation effect was significantly reduced in the −1938/−590 *CEBPD* promoter region of PC and PCmiMY (Figure 6b).

Taken together, these results suggested that miRNAs act as initiators and YY1 functions as an effector protein for the DNA methylation-mediated silencing of CEBPD gene transcription. To further confirm the importance of these miRNAs, we introduced the IPTG-inducible miR-744-, miR-3154- and miR-3162-silencing vectors into THP-1 cells. Following the induction of antagomirs of miR-744, miR-3154 and miR-3162, bortezomib-induced DNA methylation on −1938/−590 *CEBPD* region of PCmi reporter was lost (Figure 6c and Supplementary Figure S8b). Meanwhile, bortezomib-reduced cell viability and the expression of the potent oncogenes, *PRKDC*, *MCM4* and *UBE2V2*, were also significantly attenuated (Figures 6d and e). The results suggested the importance of the miR-744, miR-3154 and miR-3162-mediated autoregulation of the *CEBPD* gene and the inactivation of *PRKDC, MCM4 and UBE2V2* genes on bortezomib-induced cell viability.

## Discussion

The abundance of CEBPD could be regulated via P38/CREB pathway-upregulated transcriptional activation^21^ and RNA-binding protein HuR-stabilized *CEBPD* mRNA.^42, 43^ A previous study showed that bortezomib promotes the shuttling of HuR proteins to the cytoplasm and increases the translation efficiency of its downstream target genes in cancer cells.^44^ In addition, a recent study demonstrated that tyrosine kinase Src could downregulate CEBPD protein stability through the activation of SIAH2 E3 ubiquitin ligase.^45^ Moreover, the activation of Src kinase is inhibited by the oncoprotein BCR-ABL in leukemic cells.^46^ However, the mechanism(s) underlying bortezomib-induced CEBPD expression in leukemic cells remain an open question and require further examination.

**Figure 7:**
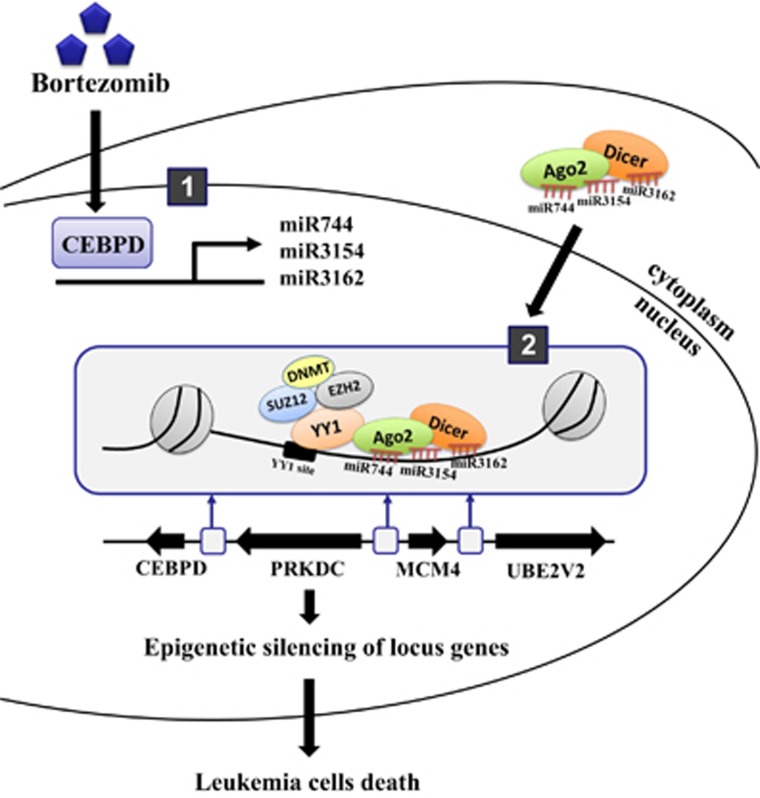
Schematic model for CEBPD turned off the locus genes and resulted in leukemic cell death through miRNA/Ago2/YY1/PcG group protein/DNMT complex-directed epigenetic silencing. In response to the anticancer drug bortezomib, the transcription factor CEBPD is activated and transcriptionally activates miR-744, miR-3154 and miR-3162. These miRNAs form a complex with Ago2 and translocate into the nucleus to target their complementary DNA sequence-binding sites on the promoter regions of CEBPD, *PRKDC*, *MCM4* and *UBE2V2*. The initiator miRNAs/Ago2 complex interacts with YY1 and recruits the epigenetic regulators, the PcG complex/DNMTs, to silence the four gene loci, including CEBPD itself. The inactivation of these potent oncogenes, *PRKDC*, *MCM4* and *UBE2V2* result in leukemic cell death via CEBPD-responsive miRNA-mediated epigenetic silencing

In this study, we found that the potent tumor suppressor CEBPD is responsive to the anticancer drug bortezomib in leukemic cells (Figure 1a). Interestingly, the inactivation of the *PRKDC*, *MCM4* and *UBE2V2* genes occurred in parallel with a decrease in *CEBPD* transcription (Supplementary Figure S4). Importantly, the inactivation of these four genes followed the increase in CEBPD-responsive miR-744, miR-3154 and miR-3162 expression (Figure 3f and Supplementary Figure S6). Here, we proposed a scenario that CEBPD, which is transiently induced by bortezomib, activates the transcription of miR-744, miR-3154 and miR-3162. Ago2 could further shuttle these three miRNAs into the nucleus and target the promoter regions of genes bearing the seed sequences complementary to these miRNAs. Moreover, the initiator miRNAs/Ago2 complex interacts with YY1 and recruits the epigenetic regulators, PcG complex/DNMTs, to inactivate the transcription of genes, including *CEBPD*, *PRKDC*, *MCM4* and *UBE2V2* (Figure 7).

MCM4 is a proliferation marker and a member of the six minichromosome maintenance^47^ proteins and is essential for the origins of DNA replication before the S phase.^48, 49^ Accumulating evidence suggests that MCM4 contributes to the proliferation of non-small-cell lung cancer cells.^50^ The *PRKDC* gene is adjacent to the *MCM4* gene on the chromosome and has a key role in non-homologous end joining.^51, 52^ The expression level of *PRKDC* was elevated in neuroblastoma patients, associated with poor prognosis and advanced tumor grade.^53^ Moreover, the DNA repair gene *UBE2V2* acts as a prognostic marker in breast cancer.^54^ A previous study suggested that attenuated UBE2V2 increases the cytotoxic effects of UV-induced DNA damage.^55^ Accumulating results have indicated that miRNAs could function as tumor suppressors in leukemia. Combined with the observations that CEBPD triggers growth arrest, participates in the bortezomib-induced cell death (Figures 1d and 2b) and activates miR-744, miR-3154 and miR-3162, we further provided a novel mechanism that miRNAs serve as tumor suppressors by eliciting DNA methylation on gDNA in leukemic cells. This study also demonstrates for the first time that CEBPD activation induces leukemic cell death by suppressing a cluster of potent oncogenes via responsive miRNA-mediated epigenetic silencing. In addition to miR-744, miR-3154 and miR-3162, several CEBPD upregulated miRNAs, including miR-150, miR-142 and miR-29a/b, were suggested to prevent leukemia progression.^56, 57, 58^ Additionally, another CEBPD-responsive miR-744 was suggested to act as a tumor suppressor in various cancers, resulting in chromosomal instability and oncogene inactivation.^59, 60^ Consistently, these CEBPD-responsive miRNAs further agree with the involvement of CEBPD in promoting cell differentiation and apoptosis.

CEBPD has been suggested to positively regulated peroxisome proliferator-activated receptor-*γ* (PPAR*γ*) transcription and protein expression in vascular smooth muscle cells and cancer cells.^37, 61, 62^ Moreover, the activation of PPAR*γ* could suppress protein expression and transcription of CEBPD via the inactivation of signal transducer and activator of transcription 3 in vascular smooth muscle cells.^61^ These findings implied that CEBPD could be negatively autoregulated following PPAR*γ* activation. In this study, we provide support for a novel feedback regulation of the CEBPD gene via miRNA-mediated autoregulation. However, the issue whether this feedback regulation occurs in tissue cells or other cancer cell needs to be examined in the future. Furthermore, significantly higher *PPARγ* mRNA was observed in primary AML patients.^63^ Therefore, in addition to revealing the contribution of epigenetic- and PPAR*γ*-mediated inactivation of CEBPD in leukemic cells, the issue of how to efficiently activate CEBPD for executing its tumor suppressor role in leukemia remains an open question.

The highly evolutionarily conserved Ago proteins bind to non-coding RNA and have an essential role in miRNA-mediated post-transcriptional regulation.^12^ Accumulating evidence has demonstrated that Ago proteins are present in the nuclear fraction of human cells. Several reports have shown that Ago proteins form a complex with lnRNA and bind to genomic DNA, thereby inducing direct TGS.^64, 65, 66, 67^ Additionally, Ago1 has been implicated in the YY1/Suz12/Dicer1 complex-mediated repression of the *NFI-A* gene via competing RNA polymerase II binding.^68^ Ago2 was also suggested to interact with the transcriptional repressor, SETDB1, for the repression of gene activation.^69^ In the present study, we provided the first evidence that Ago2, but not Ago1, Ago3 or Ago4, interacts with YY1 and binds to gDNA, and that these interactions further recruit the epigenetic regulators PcG proteins and DNMTs to direct TGS (Figure 5). The results also implied that Ago1 and Ago2 have distinct roles, at least in part, to independently contribute to PcG protein- and DNMT-mediated epigenetic regulation.

Furthermore, the PcG complex and HP-1 interact and contribute to the silencing effects at the nuclear periphery.^70, 71^ Our results showed the binding of HP-1 and the PcG complex on the promoter regions of CEBPD and its 5′ upstream genes (Figure 5c). A previous study showed that Ago1 is dispersed throughout the nuclear interior, whereas Ago2 is localized in the inner nuclear periphery, implying that this area is a transcriptionally repressive compartment.^72^ Our study raised several important issues: (1) the location of HP-1 associates with Ago1 and Ago2 distribution in the cell nucleus, (2) the location of miRNAs on gDNA associates with Ago1 and Ago2 and (3) the annotation of miRNAs associated with the cell functions in response to the epigenetic regulation. In addition to providing *in vivo* evidence associated with the physiological phenomenon, these results are consistent with those of a previous study showing that the loss of Ago2 abrogates TGS.^73^ Taken together, Ago2 not only serves as a post-transcriptional regulator via miRNA binding in the 3′-UTR but might also, at least in part, be involved in epigenetic regulation by recruiting PcG proteins and DNMT1.

## Materials and methods

### Materials

The TRIsure RNA extraction reagent, Opti-MEM medium, SuperScript III, Dulbecco’s modified Eagle's medium and Roswell Park Memorial Institute medium were purchased from Invitrogen (Carlsbad, CA, USA). Fetal bovine serum (FBS) was purchased from HyClone Laboratories (Logan, UT, USA). SensiFAST SYBR was purchased from Bioline (Taunton, MA, USA). A luciferase assay system was purchased from Promega (Madison, WI, USA). The *α*-tubulin antibody (T6199) was purchased from Sigma (St. Louis, MO, USA), and antibodies against CEBPD (SC-636), Ago2 (sc-32877), Ago3 (sc-32662), Ago4 (sc-374220) and YY1 were purchased from Santa Cruz Biotechnology (Santa Cruz, CA, USA). The anti-Ago1 antibody (no. 07-599) was purchased from Millipore (Billerica, MA, USA). The anti-SUZ12 antibody was purchased from Abcam (Cambridge, UK). The anti-DNMT1 antibody was purchased from IMGENEX (San Diego, CA, USA). All oligonucleotides were synthesized at MDBio Inc. (Taipei, Taiwan).

### Cell culture

The human monocytic leukemic cell line THP-1 and the human myeloid cell line U937 were maintained in Roswell Park Memorial Institute medium supplemented with 10% FBS, 100 *μ*g/ml streptomycin and 100 U/ml penicillin. The human embryonic kidney cell line 293T and human epithelial cells Ampho were maintained in Dulbecco’s modified Eagle's medium supplemented with 10% FBS, 100 *μ*g/ml streptomycin and 100 U/ml penicillin.

### Reverse transcription-polymerase chain reaction

THP-1 cells were treated with Dox or bortezomib. Total RNA was extracted using TRIsure. The isolated RNA was subjected to reverse transcription using SuperScript III for cDNA synthesis. The following oligonucleotide primers were used in the qPCR analysis: GAPDH (F): 5′-CCACCCAGAAGACTGTGGAT-3′ and GAPDH (R): 5′-TTCAGCTCAGGGATGACCTT-3′ CEBPD (F): 5′-GCCATGTACGACGACGAGAG-3′ and CEBPD (R): 5′-TGTGATTGCTGTTGAAGAGGTC-3′ PRKDC (F): 5′-CATGGAAGAAGATCCCCAGA-3′ and PRKDC (R): 5′-TGGGCACACCACTTTAACAA-3′ MCM4 (F): 5′-GGCTCTCATCGAGGCTTATG-3′ and MCM4 (R): 5′-TTCCACATCAATGGCTTCAA-3′ UBE2V2 (F): 5′-AAGGAGTAGGCGACGGTACA-3′ and UBE2V2 (R): 5′-ACGGAGGAGCTTCTGGGTAT-3′.

### Cell viability

For the cell survival assay, THP-1 cells were cultured on 12-well plates. The cells were grown for 24 and 48 h after treatment with bortezomib. Next, the experimental cells were incubated with diluted MTT reagent for 3 h. Subsequently, the samples were spectrophotometrically measured at 595 nm using an enzyme-linked immunosorbent assay plate reader.

### TaqMan reverse transcription-PCR for miRNA quantification

Total RNA was isolated using TRIzol according to the manufacturer’s instructions, reverse transcribed using the TaqMan microRNA Reverse Transcription Kit and subjected to real-time PCR using the TaqMan microRNA Assay Kit (Applied Biosystems, Waltham, MA, USA).

### Plasmids construction and reporter assays

The 5′-flanking region of *CEBPD*, *MAP2K4* (host gene of intronic miR744), *SNHG11* (host gene of intronic miR-3162) and miR-3154 were cloned from THP-1 cells using the DNeasy Tissue Kit (Qiagen, Düsseldorf, Germany) and PCR. The following primers were used for PCR and cloning the 5′-flanking regions: *CEBPD* (F) (−1938) *Kpn*I – 5′-GGTACCTTTGTGCTACAACTTTTTCTGG-3′ and (R) (−589) *Nhe*I – 5′-GCGAGCGCACCGCACTCGGG-3′ *MAP2K4* (F) *Kpn*I – 5′-GGGGTACCCCGATCACACAATGCTTCTAAAT-3′ and (R) *Xho*I – 5′- CCGCTCGAGCGGTGTTGGGAGTGAAGAGC-3′ *SNHG11* (F) *Kpn*I – 5′- GGGGTACCCCAGAACGCATAGCAGAAAATGTTC-3′ and (R) *Xho*I – 5′- CCGCTCGAGCGGGAGCCGCCGCCG-3′ *miR-3154* (F) *Kpn*I – 5′- GGGGTACCCCCATTTCCTGACTGCAGAGAC-3′ and (R) *Xho*I – 5′- CCGCTCGAGCGGTCCATCCAGGCAGT-3′. After verification via sequencing, the PCR products were cloned into the multicloning sites of the pGL3-promoter or pGL3-basic vector. The complete complementary sequences of mature miR744/3154/3162 were cloned into the PC reporter construct via *Nhe*I/*Hind*III restriction enzyme sites to generate PCmi. Furthermore, two YY1 binding sites on PCmi were mutated by site-directed mutagenesis using the QuikChange Site-Directed Mutagenesis Kit (Stratagene, La Jolla, CA, USA), according to the manufacturer’s instructions, to generate PCmiMY. These reporter constructs were transfected into 293T cells using TransIT-2020 transfection reagent (Mirus, Madison, WI, USA) according to the manufacturer’s instructions. The transfectants were cultured in complete medium supplemented with HA-CEBPD or bortezomib. Luciferase activity was measured in the lysates of the transfectants.

### Western blot assay

Cells were harvested and lysed in modified RIPA buffer (50 mM Tris-HCl (pH 7.4), 150 mM NaCl, 1 mM EDTA, 1% NP40, 0.25% sodium deoxycholate, 1 mM DDT, 10 mM NaF, 1 mM PSMF, 1 *μ*g/ml aprotinin, 1 *μ*g/ml leupeptin and 1 mM Na_3_VO_4_). Following lysis, the lysates were resolved by sodium dodecyl sulfate-polyacrylamide gel electrophoresis using a 12% polyacrylamide gel, and the proteins were transferred to a polyvinylidene difluoride nylon membrane and probed with primary antibodies for target proteins overnight at 4 °C. The target proteins were after incubation with peroxidase-conjugated secondary antibodies for 1 h at room temperature. The signals were revealed using an Enhanced Chemiluminescence Western Blot System (Thermo Scientific, Rockford, IL, USA).

### Chromatin immunoprecipitation

ChIP assay was conducted according to Ju-Ming Wang and co-workers.^74^ Briefly, THP-1 cells were treated with 1% formaldehyde for 15 min. The crosslinked chromatin was subsequently prepared and sonicated to an average size of 500–1000 bp. The DNA fragments were immunoprecipitated with antibodies specific for Ago1–4, SUZ12, EZH2, DNMT1, HP-1, CEBPD and control immunoglobulin G^75^ at 4 °C for 12–16 h. After the reversal of the crosslinking, the immunoprecipitated chromatin was amplified using primers specific for regions of the genomic locus of the target genes. The primers are as indicated below. MAP2K4 (F): 5′-CTCCTTTGAGGGTGTTCTGTG-3′ and (R): 5′-ACCAGGGAACAGGGCAGTA-3′ miR-3154 (F): 5′-GACTGCAGAGACCAGGAAGG-3′ and (R): 5′-CCGCTCCTACCTAGTGTCCA-3′ SNHG11 (F): 5′-TGTTTCTTTCATTCTCCAAGAATTT-3′ and (R): 5′-AAGTTTACCTGTAAGGAGAAGAACAG-3′ A (F): 5′-AGAAGTTGGTGGAGCTGTCG-3′ and (R): 5′-GGTATGGGTCGTTGCTGAGT-3′ B (F): 5′-GGCGAGACACAACGTTTTCA-3′ and (R): 5′-ATGGGGTGTATTATGGGTGT-3′ C (F): 5′-TATTTCTCTTGCCCTGCCCC-3′ and (R): 5′-GCGCTTCTCTGTGTTTAGATGA-3′ D (F): 5′-CCCCGGTTCAAACGATTCTC-3′ and (R): 5′-ATAAGTTCATGAGGGCCGGG-3′.

### Co-immunoprecipitation

The lysates of 293T cells were prepared using an immunoprecipitation lysis buffer (50 mM NaCl, 0.5% NP-40 and 10 mM Tris-HCl (pH 8.0)). The supernatant was collected and incubated with anti-YY1 antibody at 4 °C for at least 4 h. Protein-A/G agarose beads were added to the lysates, and the mixtures were incubated and rotated at 4 °C for 1 h. The beads were collected using centrifugation and washed three times with modified RIPA buffer. The proteins that were bound to the beads were eluted after adding 2 × electrophoresis sample buffer and were subsequently subjected to western blot analysis.

### Methylation-specific PCR

After treating the genomic DNA with sodium bisulfite (Zymo Research, Irvine, CA, USA), the DNA was PCR-amplified using primers specific for the methylated sequences. The primers used for the amplification of methylated and unmethylated promoters of CEBPD were designed using the MethPrimer website. The primers used were as follows: methylation (F): 5′-AGGTGTTAGAATATTTTTTTATCGA-3′ and (R): 5′-TTCCATTACACTCCAACCTAAACA-3′ nnmethylation (F): 5′-AGGTGTTAGAATATTTTTTTATTGA-3′ and (R): 5′-TTCCATTACACTCCAACCTAAACA-3′.

### Pre-miR-744/3154/3162 and antisense of miR-744/3154/3162-inducible stable cells

Pre-miR-744/3154/3162 were generated by PCR and then inserted into pAS4w.1.Pneo, a tetracycline-inducible system of lentiviral expression vector. The antisense of miR-744/3154/3162 was inserted into pLAS1w.3xLacO, a lentiviral expression vector. Stable THP-1 cells containing pre-miR-744/3154/3162 and the antisense of miR-744/3154/3162 were generated by transfecting pLAS.AS3w.aOn.Pbsd lentiviral-expressing cells and parental THP-1 cells, respectively. Dox (1 *μ*g/ml) was used to induce miR-744/3154/3162 expression. IPTG (500 *μ*M) was used to induce antisense of miR-744/3154/3162 expression. The lentiviral expression vectors were obtained from the National RNAi Core Facility located at the Genomic Research Center of Institute of Molecular Biology, Academia Sinica (Taiwan).

### Statistical analysis

All experiments were repeated at least three times, and the data were analyzed for statistical significance using Student’s *t*-test in the Prism 5 Software (La Jolla, CA, USA). The data were expressed as the means±S.E.M. Differences indicated with asterisks were considered statistically significant.

## Publisher’s Note

Springer Nature remains neutral with regard to jurisdictional claims in published maps and institutional affiliations.

## References

[bib1] He L, Hannon GJ. MicroRNAs: small RNAs with a big role in gene regulation. Nat Rev Genet 2004; 5: 522–531. 15211354 10.1038/nrg1379

[bib2] Garzon R, Marcucci G, Croce CM. Targeting microRNAs in cancer: rationale, strategies and challenges. Nat Rev Drug Discov 2010; 9: 775–789. 20885409 10.1038/nrd3179 PMC3904431

[bib3] Hammond SM, Bernstein E, Beach D, Hannon GJ. An RNA-directed nuclease mediates post-transcriptional gene silencing in *Drosophila* cells. Nature 2000; 404: 293–296. 10749213 10.1038/35005107

[bib4] Ghildiyal M, Zamore PD. Small silencing RNAs: an expanding universe. Nat Rev Genet 2009; 10: 94–108. 19148191 10.1038/nrg2504 PMC2724769

[bib5] Younger ST, Pertsemlidis A, Corey DR. Predicting potential miRNA target sites within gene promoters. Bioorg Med Chem Lett 2009; 19: 3791–3794. 19423343 10.1016/j.bmcl.2009.04.032 PMC2709707

[bib6] Gingeras TR. Origin of phenotypes: genes and transcripts. Genome Res 2007; 17: 682–690. 17567989 10.1101/gr.6525007

[bib7] de Nigris F. Epigenetic regulators: polycomb-miRNA circuits in cancer. Biochim Biophys Acta 2016; 1859: 697–704. 26975854 10.1016/j.bbagrm.2016.03.005

[bib8] Morris KV. RNA-mediated transcriptional gene silencing in human cells. Curr Top Microbiol Immunol 2008; 320: 211–224. 18268846 10.1007/978-3-540-75157-1_10

[bib9] Janowski BA, Huffman KE, Schwartz JC, Ram R, Nordsell R, Shames DS et al. Involvement of AGO1 and AGO2 in mammalian transcriptional silencing. Nat Struct Mol Biol 2006; 13: 787–792. 16936728 10.1038/nsmb1140

[bib10] Kim DH, Saetrom P, Snove O Jr., Rossi JJ. MicroRNA-directed transcriptional gene silencing in mammalian cells. Proc Natl Acad Sci USA 2008; 105: 16230–16235. 18852463 10.1073/pnas.0808830105 PMC2571020

[bib11] Liu J, Carmell MA, Rivas FV, Marsden CG, Thomson JM, Song JJ et al. Argonaute2 is the catalytic engine of mammalian RNAi. Science 2004; 305: 1437–1441. 15284456 10.1126/science.1102513

[bib12] Meister G. Argonaute proteins: functional insights and emerging roles. Nat Rev Genet 2013; 14: 447–459. 23732335 10.1038/nrg3462

[bib13] Wei Y, Li L, Wang D, Zhang CY, Zen K. Importin 8 regulates the transport of mature microRNAs into the cell nucleus. J Biol Chem 2014; 289: 10270–10275. 24596094 10.1074/jbc.C113.541417 PMC4036152

[bib14] Wang J, Lu M, Qiu C, Cui Q. TransmiR: a transcription factor-microRNA regulation database. Nucleic Acids Res 2010; 38: D119–D122. 19786497 10.1093/nar/gkp803 PMC2808874

[bib15] Chu YY, Ko CY, Wang WJ, Wang SM, Gean PW, Kuo YM et al. Astrocytic CCAAT/enhancer binding protein delta regulates neuronal viability and spatial learning ability via miR-135a. Mol Neurobiol 2016; 53: 4173–4188. 26208701 10.1007/s12035-015-9359-z PMC4937099

[bib16] Ramji DP, Foka P. CCAAT/enhancer-binding proteins: structure, function and regulation. Biochem J 2002; 365(Part 3): 561–575. 12006103 10.1042/BJ20020508 PMC1222736

[bib17] Gery S, Tanosaki S, Hofmann WK, Koppel A, Koeffler HP. C/EBPdelta expression in a BCR-ABL-positive cell line induces growth arrest and myeloid differentiation. Oncogene 2005; 24: 1589–1597. 15674331 10.1038/sj.onc.1208393

[bib18] Balamurugan K, Sterneck E. The many faces of C/EBPdelta and their relevance for inflammation and cancer. Int J Biol Sci 2013; 9: 917–933. 24155666 10.7150/ijbs.7224 PMC3805898

[bib19] Ko CY, Chang WC, Wang JM. Biological roles of CCAAT/enhancer-binding protein delta during inflammation. J Biomed Sci 2015; 22: 6. 25591788 10.1186/s12929-014-0110-2 PMC4318212

[bib20] Ko CY, Hsu HC, Shen MR, Chang WC, Wang JM. Epigenetic silencing of CCAAT/enhancer-binding protein delta activity by YY1/polycomb group/DNA methyltransferase complex. J Biol Chem 2008; 283: 30919–30932. 18753137 10.1074/jbc.M804029200 PMC2662167

[bib21] Pan YC, Li CF, Ko CY, Pan MH, Chen PJ, Tseng JT et al. CEBPD reverses RB/E2F1-mediated gene repression and participates in HMDB-induced apoptosis of cancer cells. Clin Cancer Res 2010; 16: 5770–5780. 20971808 10.1158/1078-0432.CCR-10-1025 PMC7325841

[bib22] Chuang CH, Wang WJ, Li CF, Ko CY, Chou YH, Chuu CP et al. The combination of the prodrugs perforin-CEBPD and perforin-granzyme B efficiently enhances the activation of caspase signaling and kills prostate cancer. Cell Death Dis 2014; 5: e1220. 24810056 10.1038/cddis.2014.106 PMC4047860

[bib23] Radich JP, Dai H, Mao M, Oehler V, Schelter J, Druker B et al. Gene expression changes associated with progression and response in chronic myeloid leukemia. Proc Natl Acad Sci USA 2006; 103: 2794–2799. 16477019 10.1073/pnas.0510423103 PMC1413797

[bib24] Agrawal S, Hofmann WK, Tidow N, Ehrich M, van den Boom D, Koschmieder S et al. The C/EBPdelta tumor suppressor is silenced by hypermethylation in acute myeloid leukemia. Blood 2007; 109: 3895–3905. 17234736 10.1182/blood-2006-08-040147

[bib25] Ling YH, Liebes L, Ng B, Buckley M, Elliott PJ, Adams J et al. PS-341, a novel proteasome inhibitor, induces Bcl-2 phosphorylation and cleavage in association with G2–M phase arrest and apoptosis. Mol Cancer Ther 2002; 1: 841–849. 12492117

[bib26] Kouroukis TC, Baldassarre FG, Haynes AE, Imrie K, Reece DE, Cheung MC. Bortezomib in multiple myeloma: systematic review and clinical considerations. Curr Oncol 2014; 21: e573–e603. 25089109 10.3747/co.21.1798 PMC4117625

[bib27] Kelley TW, Alkan S, Srkalovic G, Hsi ED. Treatment of human chronic lymphocytic leukemia cells with the proteasome inhibitor bortezomib promotes apoptosis. Leuk Res 2004; 28: 845–850. 15203282 10.1016/j.leukres.2003.12.010

[bib28] Zheng RP, Wang W, Wei CD. Bortezomib inhibits cell proliferation in prostate cancer. Exp Ther Med 2015; 10: 1219–1223. 26622468 10.3892/etm.2015.2617 PMC4533241

[bib29] Nawrocki ST, Carew JS, Pino MS, Highshaw RA, Dunner K Jr., Huang P et al. Bortezomib sensitizes pancreatic cancer cells to endoplasmic reticulum stress-mediated apoptosis. Cancer Res 2005; 65: 11658–11666. 16357177 10.1158/0008-5472.CAN-05-2370

[bib30] Hong YS, Hong SW, Kim SM, Jin DH, Shin JS, Yoon DH et al. Bortezomib induces G2-M arrest in human colon cancer cells through ROS-inducible phosphorylation of ATM-CHK1. Int J Oncol 2012; 41: 76–82. 22552540 10.3892/ijo.2012.1448

[bib31] Schnerch D, Schuler J, Follo M, Felthaus J, Wider D, Klingner K et al. Proteasome inhibition enhances the efficacy of volasertib-induced mitotic arrest in AML *in vitro* and prolongs survival *in vivo*. Oncotarget 2017; 8: 21153–21166. 28416751 10.18632/oncotarget.15503 PMC5400573

[bib32] Mannava S, Zhuang D, Nair JR, Bansal R, Wawrzyniak JA, Zucker SN et al. KLF9 is a novel transcriptional regulator of bortezomib- and LBH589-induced apoptosis in multiple myeloma cells. Blood 2012; 119: 1450–1458. 22144178 10.1182/blood-2011-04-346676 PMC3286209

[bib33] de Wilt LH, Kroon J, Jansen G, de Jong S, Peters GJ, Kruyt FA. Bortezomib and TRAIL: a perfect match for apoptotic elimination of tumour cells? Crit Rev Oncol Hematol 2013; 85: 363–372. 22944363 10.1016/j.critrevonc.2012.08.001

[bib34] Zhang L, Sui Y, Wang T, Li L, Li Y, Jin C et al. Roles of hMMS2 gene in reversing the oxaliplatin tolerance of human colon carcinoma cells. Yi chuan = Hereditas 2014; 36: 346–353. 24846979

[bib35] Maiorano D, Van Assendelft GB, Kearsey SE. Fission yeast cdc21, a member of the MCM protein family, is required for onset of S phase and is located in the nucleus throughout the cell cycle. EMBO J 1996; 15: 861–872. 8631307 PMC450284

[bib36] Stronach EA, Chen M, Maginn EN, Agarwal R, Mills GB, Wasan H et al. DNA-PK mediates AKT activation and apoptosis inhibition in clinically acquired platinum resistance. Neoplasia 2011; 13: 1069–1080. 22131882 10.1593/neo.111032 PMC3223610

[bib37] Li CF, Tsai HH, Ko CY, Pan YC, Yen CJ, Lai HY et al. HMDB and 5-AzadC combination reverses tumor suppressor CCAAT/enhancer-binding protein delta to strengthen the death of liver cancer cells. Mol Cancer Ther 2015; 14: 2623–2633. 26358750 10.1158/1535-7163.MCT-15-0025

[bib38] Chi JY, Hsiao YW, Li CF, Lo YC, Lin ZY, Hong JY et al. Targeting chemotherapy-induced PTX3 in tumor stroma to prevent the progression of drug-resistant cancers. Oncotarget 2015; 6: 23987–24001. 26124179 10.18632/oncotarget.4364 PMC4695165

[bib39] Wang WJ, Li CF, Chu YY, Wang YH, Hour TC, Yen CJ et al. Inhibition of the EGFR/STAT3/CEBPD axis reverses cisplatin cross-resistance with paclitaxel in the urothelial carcinoma of the urinary bladder. Clin Cancer Res 2017; 23: 503–513. 27435393 10.1158/1078-0432.CCR-15-1169

[bib40] Holoch D, Moazed D. RNA-mediated epigenetic regulation of gene expression. Nat Rev Genet 2015; 16: 71–84. 25554358 10.1038/nrg3863 PMC4376354

[bib41] Carthew RW, Sontheimer EJ. Origins and mechanisms of miRNAs and siRNAs. Cell 2009; 136: 642–655. 19239886 10.1016/j.cell.2009.01.035 PMC2675692

[bib42] Li B, Si J, DeWille JW. Ultraviolet radiation (UVR) activates p38 MAP kinase and induces post-transcriptional stabilization of the C/EBPdelta mRNA in G0 growth arrested mammary epithelial cells. J Cell Biochem 2008; 103: 1657–1669. 17902160 10.1002/jcb.21554

[bib43] Hsiao YW, Li CF, Chi JY, Tseng JT, Chang Y, Hsu LJ et al. CCAAT/enhancer binding protein delta in macrophages contributes to immunosuppression and inhibits phagocytosis in nasopharyngeal carcinoma. Sci Signal 2013; 6: ra59. 23861541 10.1126/scisignal.2003648

[bib44] Kandasamy K, Kraft AS. Proteasome inhibitor PS-341 (VELCADE) induces stabilization of the TRAIL receptor DR5 mRNA through the 3'-untranslated region. Mol Cancer Ther 2008; 7: 1091–1100. 18483298 10.1158/1535-7163.MCT-07-2368 PMC3632452

[bib45] Sarkar TR, Sharan S, Wang J, Pawar SA, Cantwell CA, Johnson PF et al. Identification of a Src tyrosine kinase/SIAH2 E3 ubiquitin ligase pathway that regulates C/EBPdelta expression and contributes to transformation of breast tumor cells. Mol Cell Biol 2012; 32: 320–332. 22037769 10.1128/MCB.05790-11 PMC3255785

[bib46] Amarante-Mendes GP, Naekyung Kim C, Liu L, Huang Y, Perkins CL, Green DR et al. Bcr-Abl exerts its antiapoptotic effect against diverse apoptotic stimuli through blockage of mitochondrial release of cytochrome C and activation of caspase-3. Blood 1998; 91: 1700–1705. 9473236

[bib47] Poulaki V, Mitsiades CS, Kotoula V, Negri J, McMillin D, Miller JW et al. The proteasome inhibitor bortezomib induces apoptosis in human retinoblastoma cell lines *in vitro*. Invest Ophthalmol Vis Sci 2007; 48: 4706–4719. 17898295 10.1167/iovs.06-1147

[bib48] Tye BK. MCM proteins in DNA replication. Annu Rev Biochem 1999; 68: 649–686. 10872463 10.1146/annurev.biochem.68.1.649

[bib49] Kearsey SE, Maiorano D, Holmes EC, Todorov IT. The role of MCM proteins in the cell cycle control of genome duplication. BioEssays 1996; 18: 183–190. 8867732 10.1002/bies.950180305

[bib50] Kikuchi J, Kinoshita I, Shimizu Y, Kikuchi E, Takeda K, Aburatani H et al. Minichromosome maintenance (MCM) protein 4 as a marker for proliferation and its clinical and clinicopathological significance in non-small cell lung cancer. Lung Cancer 2011; 72: 229–237. 20884074 10.1016/j.lungcan.2010.08.020

[bib51] Burma S, Chen DJ. Role of DNA-PK in the cellular response to DNA double-strand breaks. DNA Rep 2004; 3: 909–918. 10.1016/j.dnarep.2004.03.021 15279776

[bib52] Zha S, Jiang W, Fujiwara Y, Patel H, Goff PH, Brush JW et al. Ataxia telangiectasia-mutated protein and DNA-dependent protein kinase have complementary V(D)J recombination functions. Proc Natl Acad Sci USA 2011; 108: 2028–2033. 21245310 10.1073/pnas.1019293108 PMC3033273

[bib53] Dolman ME, van der Ploeg I, Koster J, Bate-Eya LT, Versteeg R, Caron HN et al. DNA-dependent protein kinase as molecular target for radiosensitization of neuroblastoma cells. PLoS ONE 2015; 10: e0145744. 26716839 10.1371/journal.pone.0145744 PMC4696738

[bib54] Santarpia L, Iwamoto T, Di Leo A, Hayashi N, Bottai G, Stampfer M et al. DNA repair gene patterns as prognostic and predictive factors in molecular breast cancer subtypes. Oncologist 2013; 18: 1063–1073. 24072219 10.1634/theoncologist.2013-0163 PMC3805146

[bib55] Li Z, Xiao W, McCormick JJ, Maher VM. Identification of a protein essential for a major pathway used by human cells to avoid UV-induced DNA damage. Proc Natl Acad Sci USA 2002; 99: 4459–4464. 11917106 10.1073/pnas.062047799 PMC123670

[bib56] Xiao C, Calado DP, Galler G, Thai TH, Patterson HC, Wang J et al. MiR-150 controls B cell differentiation by targeting the transcription factor c-Myb. Cell 2007; 131: 146–159. 17923094 10.1016/j.cell.2007.07.021

[bib57] Yendamuri S, Calin GA. The role of microRNA in human leukemia: a review. Leukemia 2009; 23: 1257–1263. 19148134 10.1038/leu.2008.382

[bib58] Xu XD, Wu XH, Fan YR, Tan B, Quan Z, Luo CL. Exosome-derived microRNA-29c induces apoptosis of BIU-87 cells by down regulating BCL-2 and MCL-1. Asian Pacific J Cancer Prev 2014; 15: 3471–3476. 10.7314/apjcp.2014.15.8.3471 24870742

[bib59] Huang V, Place RF, Portnoy V, Wang J, Qi Z, Jia Z et al. Upregulation of cyclin B1 by miRNA and its implications in cancer. Nucleic Acids Res 2012; 40: 1695–1707. 22053081 10.1093/nar/gkr934 PMC3287204

[bib60] Lin F, Ding R, Zheng S, Xing D, Hong W, Zhou Z et al. Decrease expression of microRNA-744 promotes cell proliferation by targeting c-Myc in human hepatocellular carcinoma. Cancer Cell Int 2014; 14: 58. 24991193 10.1186/1475-2867-14-58 PMC4079640

[bib61] Takata Y, Kitami Y, Yang ZH, Nakamura M, Okura T, Hiwada K. Vascular inflammation is negatively autoregulated by interaction between CCAAT/enhancer-binding protein-delta and peroxisome proliferator-activated receptor-gamma. Circ Res 2002; 91: 427–433. 12215492 10.1161/01.res.0000031271.20771.4f

[bib62] Lai PH, Wang WL, Ko CY, Lee YC, Yang WM, Shen TW et al. HDAC1/HDAC3 modulates PPARG2 transcription through the sumoylated CEBPD in hepatic lipogenesis. Biochim Biophys Acta 2008; 1783: 1803–1814. 18619497 10.1016/j.bbamcr.2008.06.008

[bib63] Tabe Y, Konopleva M, Andreeff M, Ohsaka A. Effects of PPARgamma ligands on leukemia. PPAR Res 2012; 2012: 483656. 22685453 10.1155/2012/483656 PMC3364693

[bib64] Chu Y, Yue X, Younger ST, Janowski BA, Corey DR. Involvement of argonaute proteins in gene silencing and activation by RNAs complementary to a non-coding transcript at the progesterone receptor promoter. Nucleic Acids Res 2010; 38: 7736–7748. 20675357 10.1093/nar/gkq648 PMC2995069

[bib65] Huang V, Zheng J, Qi Z, Wang J, Place RF, Yu J et al. Ago1 Interacts with RNA polymerase II and binds to the promoters of actively transcribed genes in human cancer cells. PLoS Genet 2013; 9: e1003821. 24086155 10.1371/journal.pgen.1003821 PMC3784563

[bib66] Weinmann L, Hock J, Ivacevic T, Ohrt T, Mutze J, Schwille P et al. Importin 8 is a gene silencing factor that targets argonaute proteins to distinct mRNAs. Cell 2009; 136: 496–507. 19167051 10.1016/j.cell.2008.12.023

[bib67] Robb GB, Brown KM, Khurana J, Rana TM. Specific and potent RNAi in the nucleus of human cells. Nat Struct Mol Biol 2005; 12: 133–137. 15643423 10.1038/nsmb886

[bib68] Zardo G, Ciolfi A, Vian L, Starnes LM, Billi M, Racanicchi S et al. Polycombs and microRNA-223 regulate human granulopoiesis by transcriptional control of target gene expression. Blood 2012; 119: 4034–4046. 22327224 10.1182/blood-2011-08-371344

[bib69] Cho S, Park JS, Kang YK. AGO2 and SETDB1 cooperate in promoter-targeted transcriptional silencing of the androgen receptor gene. Nucleic Acids Res 2014; 42: 13545–13556. 25183519 10.1093/nar/gku788 PMC4267665

[bib70] Beisel C, Paro R. Silencing chromatin: comparing modes and mechanisms. Nat Rev Genet 2011; 12: 123–135. 21221116 10.1038/nrg2932

[bib71] Shevelyov YY, Nurminsky DI. The nuclear lamina as a gene-silencing hub. Curr Issues Mol Biol 2012; 14: 27–38. 21795760

[bib72] Ahlenstiel CL, Lim HG, Cooper DA, Ishida T, Kelleher AD, Suzuki K. Direct evidence of nuclear Argonaute distribution during transcriptional silencing links the actin cytoskeleton to nuclear RNAi machinery in human cells. Nucleic Acids Res 2012; 40: 1579–1595. 22064859 10.1093/nar/gkr891 PMC3287199

[bib73] Ross JP, Kassir Z. The varied roles of nuclear argonaute-small RNA complexes and avenues for therapy. Mol Ther Nucleic Acids 2014; 3: e203. 25313622 10.1038/mtna.2014.54 PMC4217078

[bib74] Wang WL, Lee YC, Yang WM, Chang WC, Wang JM. Sumoylation of LAP1 is involved in the HDAC4-mediated repression of COX-2 transcription. Nucleic Acids Res 2008; 36: 6066–6079.. 10.1093/nar/gkn607 PMC2577330 18820298

[bib75] Osterhout DJ, Frazier WA, Higgins D. Thrombospondin promotes process outgrowth in neurons from the peripheral and central nervous systems. Dev Biol 1992; 150: 256–265. 1551474 10.1016/0012-1606(92)90240-h

