# Supplementary material for: Bortezomib-induced miRNAs direct epigenetic silencing of locus genes and trigger apoptosis in leukemia
Source: Cell Death Dis. 2017 Nov 9;8(11):e3167–. doi: 10.1038/cddis.2017.520 (PMC5775404; doi:10.1038/cddis.2017.520)
Supplement: Supplementary Information [file cddis2017520x1.pdf]

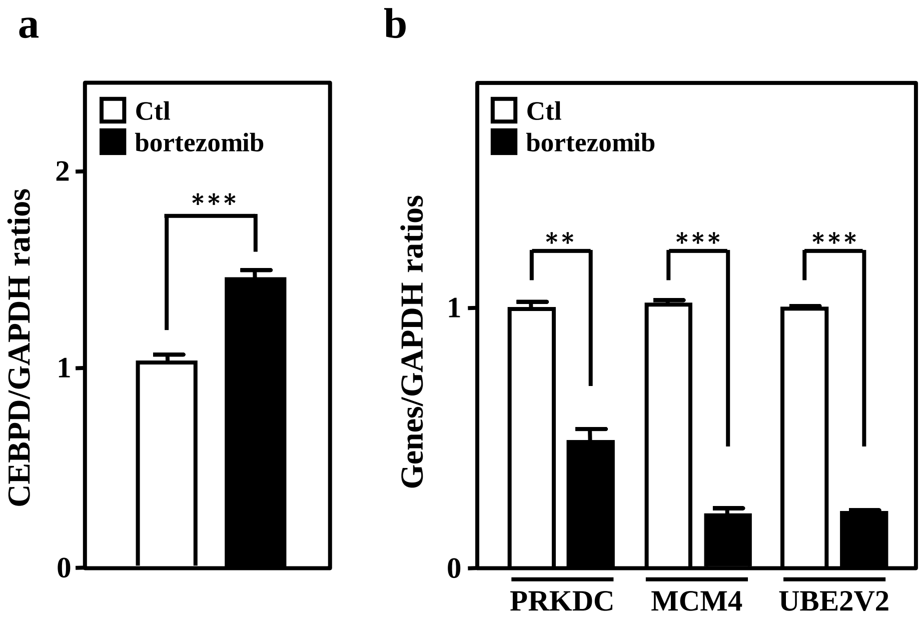

Supplementary Figure S1 Bortezomib contributes to *CEBPD* transcriptional activation and inhibits the expression of *PRKDC*, *MCM4* and *UBE2V2* genes in U937 cells. After treating with bortezomib (50 nM) for 3 h (**a**) or 16 h (**b**) in U937 cells, total RNA was harvested and qPCR was performed to check the expression of *CEBPD* or its 5'-flanking genes (*PRKDC*, *MCM4* and *UBE2V2*). The data represented the mean ± standard error of experiments performed in triplicate. (**$p < 0.01$, ***$p < 0.001$, Student's *t*-test).

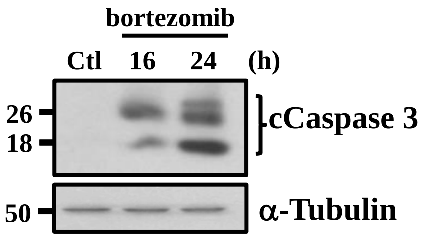

Supplementary Figure S2 Bortezomib increases the signals of activated caspase 3 in leukemia cells. After bortezomib treatment for 16 h and 24 h in THP-1 cells, protein lysates were harvested to perform Western blot analysis.

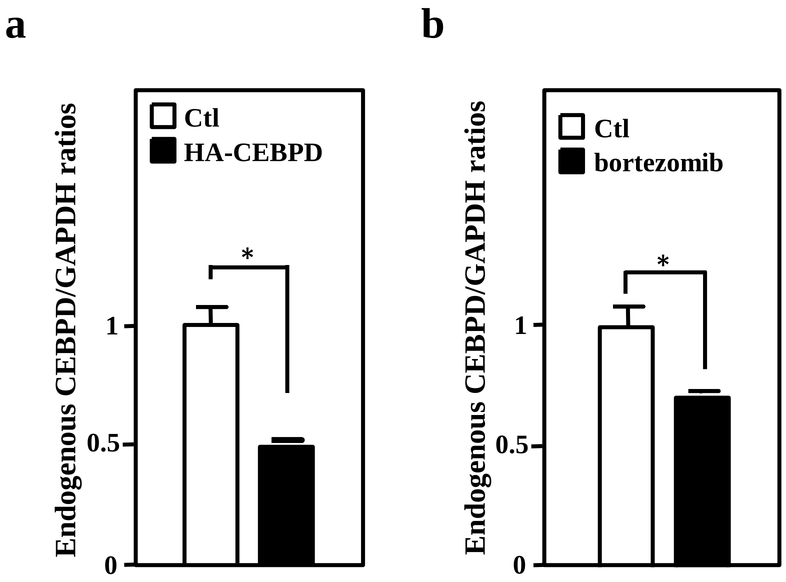

Supplementary Figure S3 Exogenous expression of HA-CEBPD and long term bortezomib treatment attenuate endogenous CEBPD transcripts. THP-1 cells (**a**) were transfected with HA-CEBPD for 18 h or (**b**) treated with bortezomib (50 nM) for 18 h. Total RNA was harvested and qPCR was used to analyze the expression of CEBPD. The data represented the mean ± standard error of experiments performed in triplicate. (*$p < 0.05$, Student's *t*-test).

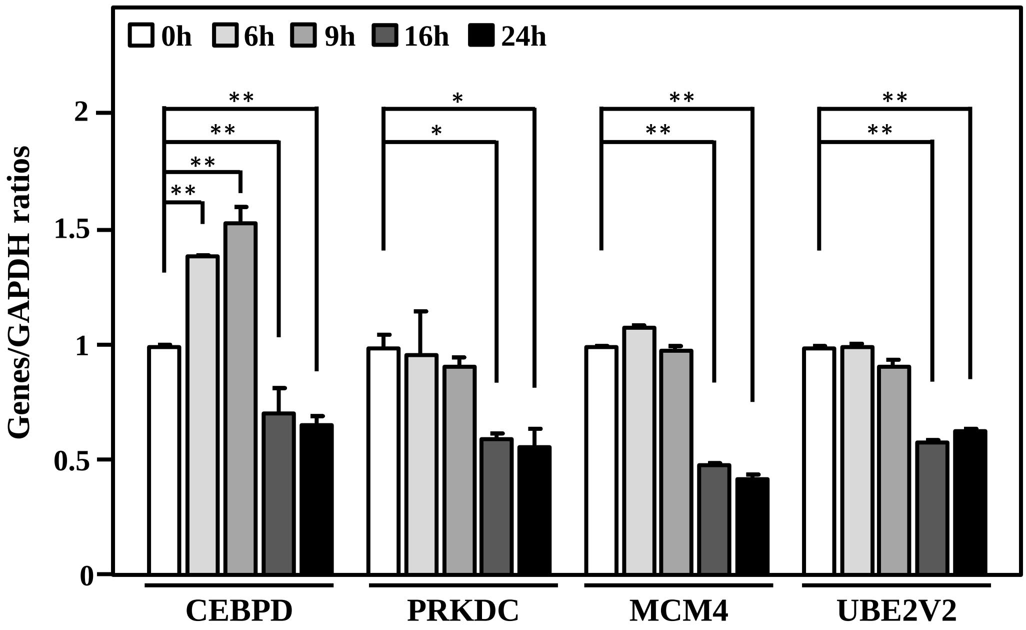

Supplementary Figure S4 PRKDC, MCM4 and UBE2V2 genes are attenuated in parallel with the decrease in CEBPD transcription upon bortezomib treatment. THP-1 cells were treated with bortezomib (50 nM). Total RNA was harvested at the indicated time points and qPCR was used to analyze the expression of genes (*CEBPD*, *PRKDC*, *MCM4*, and *UBE2V2*). The data represented the mean ± standard error of experiments performed in triplicate. (*$p < 0.05$, **$p < 0.01$, Student's *t*-test).

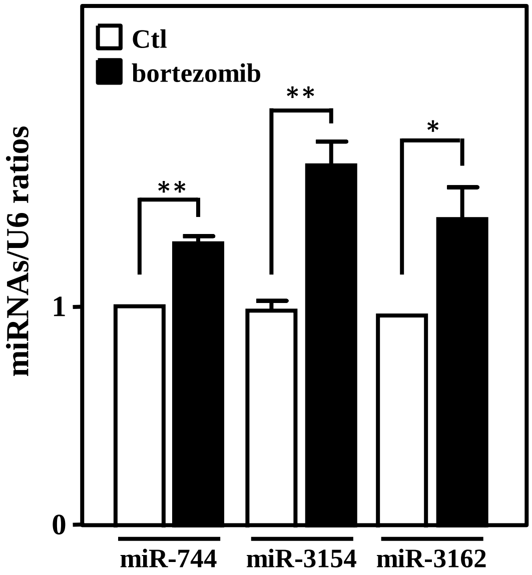

Supplementary Figure S5 Bortezomib increases miR-744, miR-3154 and miR-3162 expression. qPCR was performed using total RNA harvested from U937 cells treated with bortezomib (50 nM) for 6 h. The data represented the mean ± standard error of experiments performed in triplicate. (*$p < 0.05$, **$p < 0.01$, Student's *t*-test).

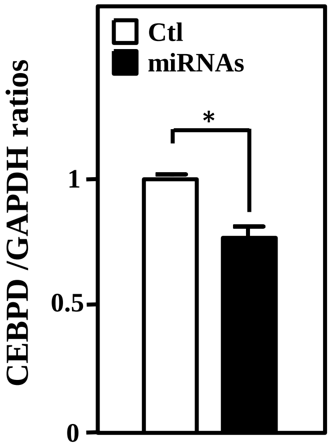

Supplementary Figure S6 Exogenous miR-744, miR-3154 and miR-3162 suppress the transcription of CEBPD. THP-1 cells were co-transfected with miR-744, miR-3154 and miR-3162 expression vectors for 18 h. Total RNA was harvested and qPCR was used to analyze the expression of CEBPD. The data represented the mean ± standard error of experiments performed in triplicate. (*p < 0.05, Student's t-test).

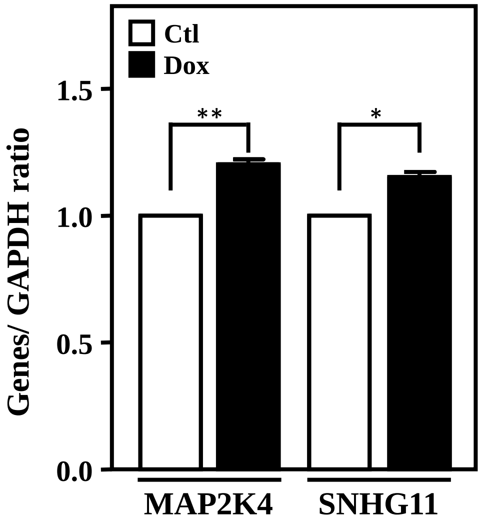

Supplementary Figure S7 CEBPD induces *MAP2K4* and *SKHG11* expression. After Dox (1 ug/ml) treatment in THP-1 cells with HA-CEBPD inducible system for 3 h, total RNA was harvested to perform the qPCR analysis. The data represented the mean ± standard error of experiments performed in triplicate. (*$p < 0.05$, **$p < 0.01$, Student's *t*-test).

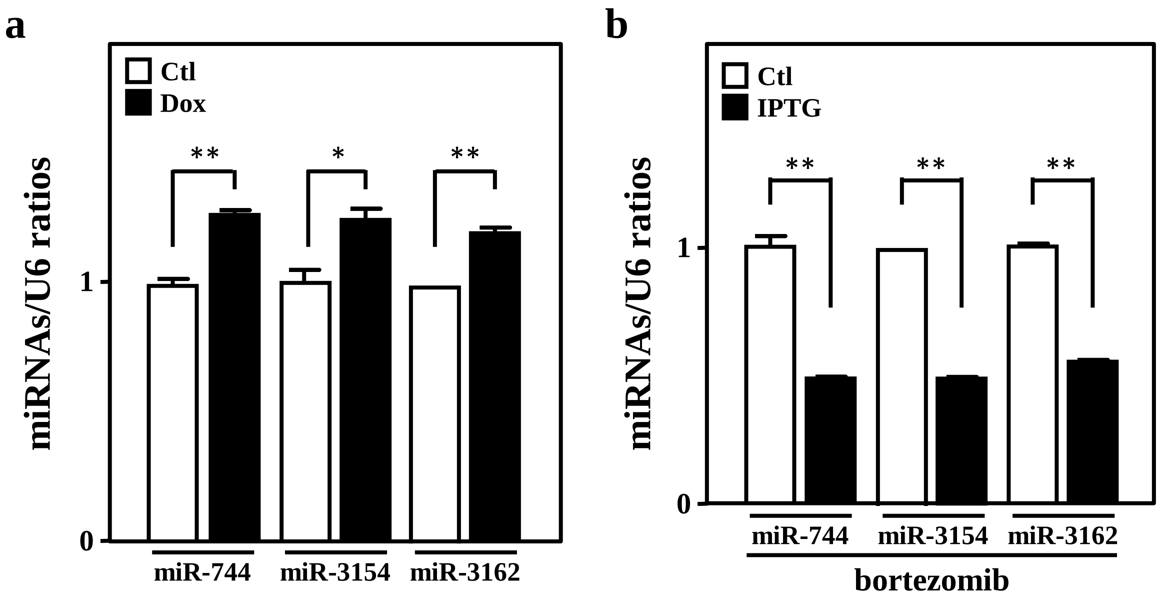

Supplementary Figure S8 miR-744, miR-3154 and miR-3162 levels are checked in Dox-inducible or IPTG inducible-silencing system. (a) After Dox treatment for 24 h, total RNA was harvested, and the levels of miRNAs (miR-744, miR-3154 and miR-3162) were confirmed by qPCR. (b) THP-1 cells with the IPTG-inducible silencing miR-744/3154/3162 system were pre-treated with IPTG (500 μM) for 3 h. After bortezomib (50 nM) for 16 h, the expression levels of miR-744, miR-3154 and miR-3162 were measured by qPCR. The data represented the mean ± standard error of experiments performed in triplicate. (**p* < 0.05, ***p* < 0.01, Student's *t*-test).

Supplementary Table 1 CEBPD down-regulated mRNAs (fold<1.5)

CCL14	lnc-SNTG1-1	lnc-AKIP1-2	ZFP42
XLOC_l2_010691	FNL3	lnc-GEMIN2-2	lnc-HMCN1-2
KRT40	lnc-HSPB11-1	XLOC_l2_015075	FAM150A
LOC100422737	lnc-RPL30-2	lnc-DRD5-8	TFEC-2
LOC100134423	lncC2CD4A-7	lncERG-1	SIK1-4
lncARL4A-2	lncAC009365.3.1-4	GADD45G	MAGEE1
lncNADSYN12	ABCA9-AS1	XLOC_l2_011098	EFHD1
LOC729970	LINC01339	LINC00222	SBSN
LOC101928697	lnc-NAV1-1	FAM110B	NAV2-AS5
DCAF8L1	lnc-CNIH-1	LOC101926928	APOL3
XLOC_l2_012107	lnc-UBAP2-1	LOC101927389	lnc-TAB3-1
H2BFWT	lnc-ZNF675-1	lnc-RAB40B-1	MED15P9
FAM189A2	lnc-BTG3-2	lnc-TPCN2-3	lnc-RUNX2-1
lnc-GREM2-8	lnc-FAM131B-1	LINC01501	IFNA7
KLK14	LOC101929294	LOC101929073	lnc-FMN2-1
C12orf54	TMEM219	lnc-NUMB-2	lnc-CCT4-1
lncGABARAPL3-2	lnc-BTNL8-2	LINC01146	DNAH10
LINC01021	FER1L6	lnc-GABRA5-4	lnc-MARCH7-2
SMAD1-AS1	lnc-SLC38A2-1	LOC101928674	lnc-BSND-1
lnc-ALG14-2	lnc-TAX1BP3-1	lnc-VAPB-1	lnc-EIF2AK3-1
LOC101928117	lnc-NVL-3	THPO;PTK2	RGS4
CD72	SNORD112	lnc-SULF1-1	CXCL3
lnc-TNFRSF19-5	lncRP11-791J7.2.1-1	lnc-LY86-3	XLOC_l2_013547
lnc-IWS1-1	LPP-AS1	lnc-GPRC5A-2	lnc-PLEKHA5-4
lnc-BCHE-1	BMP3	MIAT	lnc-KHDRBS3-5
lnc-TRIB1-3	LOC101929284	LOC102724973	lnc-CR392000.1-1
MYO16-AS1	LOC401134	LINC01257	lnc-RGL4-4
lncEXOC2-5	lncTTC33-1	LINC01305	ASB11

OR52B2	XLOC_l2_000399	XLOC_l2_001196	lnc-TUFM-3
LOC399886	XLOC_l2_007237	TSC22D1-AS1	lnc-ANP32A-3
lnc-TRIML2-3	C18orf61	KLHL34	TCN1
lncRP11-410N8.4.1-1	lnc-LEPROT-1	lnc-UTP11L-4	lnc-SLC2A4RG-1
lnc-TAS2R42-1	MGAM	SHISA9-2	AGAP2-AS1
USP43	lnc-RP11-680F20.6.1-1	lnc-DHX37-7	QRICH2
XLOC_l2_014245	HID1	SPPL2B	ALKBH8
lnc-TXNRD1-1	XLOC_l2_015098	XLOC_l2_009889	ACP5
MAMDC2-AS1	lnc-PDGFA-2;	LINC00664	XLOC_l2_007449
LOC100996890	lnc-C14orf118-1	LIPE	lnc-NDUFS5-2
lnc-NACA2-2	lnc-LTN1-1	LOC102606466	LOC101927132
LOC644083	lnc-SFN-1	lnc-ABT1-4	lnc-HOXC4-4
lnc-AC092327.1-3	lnc-DIAPH1-2	lnc-GIT1-1	lnc-RTL1-6
FAM41C	ZNF442	ZACN	KIAA1958
lnc-IL17B-3	lnc-C19orf26-2	lnc-ZNF516-4	DUSP9
IL26	lnc-SYNM-2	TTC39A-AS1	LOC101927604
PTCHD1-AS	FAM198A	SMKR1	lnc-TFB2M-2
lnc-PPFIA2-1	lnc-BCAN-1	XLOC_l2_013328	FAM83A
lncENDOD1-1	LOC101928106	LOC100129034	lnc-ZNF572-1
NRG3AS1	lnc-PRKAA2-1	lnc-LGALS14-1	lnc-GPT2-1
A2MP1	PADI6	NIPAL4	COL24A1
PIGC	HCFC1-AS1	lnc-TOR2A-1	EFCC1
APOL5	lnc-LPIN3-1	LOC101927389	lnc-MFSD9-4
LOC101929261	LOC255654	LOC101928424	lnc-RRP8-1
LOC102724712	KIAA1671	VWA5B1	lnc-POTEB-5
LOC283332	lnc-C5orf63-3	lnc-SLC29A4-1	UNC5C
LINC00518	lnc-RAMP3-2	FAAHP1	lnc-FBXO7-1
SNORA19	lnc-COL19A1-1	OR7D2	LOC102723481

FAM182B	LOC102724861	KLK1	NCAN
lnc-MYB-3	lnc-AC092117	SMN2	LOC100507494
lnc-NKX2-5-2	lnc-DRD5-3	TDRD3	LINC00243
TMEM262	LOC100506801	lnc-RP11-728F	LOC100289455
OR2G2	ZNF713	IQSEC3	SNORA23
lnc-HRH4-9	LOC100128591	ZBTB37	ASAH2
lnc-STX2-6	TNXB	lnc-CCDC23-1	LINC01550
MFAP5	lnc-SELV.1-1	lnc-ATG16L2-4	BAI2
lnc-CALCOCO2-5	lnc-FAM82A1-2	LOC101927187	WDR91
lnc-IKZF2-2	lnc-POTEB-4	lnc-GPCPD1-1	RFPL3S
lnc-ITSN1-2	LOC100131907	LOC100507420	HCRP1
FEZF1-AS1	lncCA8-2	KAZN	LOC101928108
FAM78B	lncEIF3A-1	ZNF174	SPTSSB
FLJ46363	LOC283038	lnc-JMJD4-1	XLOC_l2_015830
lnc-C20orf96-4	XLOC_l2_012844	lnc-C2orf63-1	lnc-MLL3-1
lnc-SYK-3	UXT-AS1	lnc-PTGS1-1	lnc-AGRP-3
LOC283140	lnc-ZNF536-2	lnc-ROM1-2	ANKRD20A12P
ART5	LOC101929709	lnc-ANTXRL-2	lnc-FSCN2-1
lncGIP-1	lnc-XRN2-3	TP53TG3C	lnc-CTXN1-1
LINC00957	LOC101928701	TTTY22	lnc-MRPS9-2
MTERF4	lnc-BCL3-1	lnc-FRG1-4	lnc-BMP7-2
GABRG2	lnc-CRIPAK-1	lnc-SLCO2B1-2	YAP1
XLOC_l2_005020	MRPS31P5	lnc-XRCC3-1	lnc-CD207-1
SP6	lnc-CENPV-2	lnc-AP3M1-1	TP53TG3
FOXA3	SVILP1	SNORA75	GSTTP2
ZXDA	TRDMT1	lncTCTN3-1	MAFG-AS1
XLOC_l2_006832	LOC102725053	XLOC_l2_013467	LOC100129620
MDN1	lnc-CACNA1E-1	GDNF	FAM86JP

FAM182B	XLOC_l2_008755	lnc-TIGD7-1
ACTL8	C2CD4D	ZBED6
LOC145837	LIMS3-LOC440895	FURIN
lnc-LRRTM1-4	lnc-AK1-1	LOC100996249
LCN8	CD84	FAHD2A
GHITM	lnc-WDR33-1	LOC100130930
IQCF3	R3HDM1	TRAF3IP2-AS1
CD247	lnc-GSDMD-3	UNC13B
LOC101929450	XLOC_l2_009887	SNHG4
lnc-MAPK13-1	lnc-BRD1-3	lnc-C2-2
SLC35G5	lnc-SNX33-1	lnc-TUFM-1
CECR5AS1	EPHB4	CCL2
LOC100130370	lnc-LGALSL-1	lnc-GLYCTK-1
LOC100130992	lnc-CARHSP1-1	
LOC101929260	lnc-LTBP3-2	
DDX3Y	CUX1	
FOLR2	LOC101928223	
LOC101927187	DTWD2	
XLOC_l2_009510	OR5A1	
NR4A2	lnc-UCHL3-1	
LOC102724601	LOC100129931	
KCNIP2-AS1	ND4L	
XLOC_l2_010511	LINC00092	
lnc-EXT1-2	C15orf59	
PIP5KL1	lnc-ARID2-3	
KHDRBS3	lnc-C1QTNF8-4	
ADAMTS13	lnc-SH2D7-5	
lnc-CCDC117-1	lnc-SOX6-1	

Supplementary Table 2 CEBPD up-regulated miRNAs (fold >1.2)

Name	fold	Name	fold
hsa-miR-146a	2.840208645	hsa-miR-4271	1.306660292
hsa-miR-744	2.106482675	hsa-miR-612	1.301773568
hsa-miR-933	1.627204569	hsa-miR-132	1.293610649
hsa-miR-92a-2*	1.603911206	hsa-miR-92b*	1.291836835
hsa-miR-3162	1.595569915	hsa-miR-1228*	1.283250254
hsa-miR-513c	1.574166541	hsa-miR-658	1.281080506
hsa-miR-149*	1.546803937	hsa-miR-30a	1.277662919
hsa-miR-939	1.509907844	hsa-miR-1915	1.275511776
hsa-miR-762	1.500715455	hsa-miR-542-5p	1.272804248
hsa-miR-513a-5p	1.487809038	hsa-miR-509-3p	1.266211636
hsa-miR-4270	1.474365813	hsa-miR-1249	1.25650319
hsa-miR-1207-5p	1.45695961	hsa-miR-30c-2*	1.254980699
hsa-miR-3154	1.438149847	hsa-miR-1182	1.250235642
hsa-miR-4294	1.410622963	hsa-miR-30b*	1.250196982
hsa-miR-30e	1.398637323	hsa-miR-663b	1.237967817
hsa-miR-1909	1.394264791	hsa-miR-3156	1.233676511
hsa-miR-665	1.38588901	hsa-miR-1275	1.231502023
hsa-miR-1246	1.370431657	hsa-miR-650	1.230442177
hsa-miR-130b	1.362349926	hsa-miR-320e	1.224376136
hsa-miR-1290	1.361496976	hsa-miR-1268	1.223864649
hsa-miR-1303	1.359036833	hsa-miR-3195	1.223724846
hsa-miR-513b	1.355428823	hsa-miR-3131	1.215899386
hsa-miR-765	1.350555668	hsa-miR-3177	1.214808724
hsa-miR-483-5p	1.3499965	hsa-miR-711	1.214726869
hsa-miR-936	1.345067292	hsa-miR-23a*	1.21053193
hsa-miR-493	1.337485937	hsa-miR-1972	1.208032741
hsa-miR-1224-3p	1.336405389	hsa-miR-675	1.207100933
hsa-miR-3196	1.323616556	hsa-miR-920	1.206839033
hsa-miR-557	1.316007867	hsa-miR-877	1.202246799
hsa-miR-638	1.311363949	hsa-miR-4299	1.201790342
hsa-miR-494	1.309129272		

Supplementary Table 3 The pairing of mRNAs and miRNAs regulated by CEBPD

Gene	miRNA	Gene	miRNA
SOX30	miR3154	ZNF680	miR3162
LRRTM3	miR3154, miR1285	C8orf22	miR146a
TAT	miR3154, miR665, miR708	HHATL	miR1275
PDGFRB	miR3154, miR665, miR1285, miR3162, miR1275, miR4271, miR4271, miR744	CHST10	miR513a, miR665
HKDC1	miR665	BGN	miR1275, miR422a, miR3154, miR4274
CNTNAP5	miR665	MAGIX	miR658, miR3177
IGSF11	miR665, miR1275, miR513a, miR4271	EPHB2	miR513a, miR665, miR744, miR4271
CELF3	miR1285, miR4271, miR1203, miR422a	BZRAP1	miR1275, miR422a, miR2861, miR3154
LOC200726	miR455	XRRA1	miR1285, miR665
TMTC3	miR3162	PDZD3	miR1275, miR1285, miR744, miR4271
CA6	mir92b	CROCC	miR1203, miR1275, miR1285, miR422a, miR513a, miR708, miR744, miR3154
HNRNPA2B1	miR3154, miR3162	SLC25A2	miR3154
TMEM132C	miR141, miR658	HSD11B2	miR665, miR1275
SYNJ2	miR141, miR4271	PRRG2	miR744
RASL10B	miR1285. miR3154, miR4271	TLN1	miR744, miR4271
LIN28A	miR3154, miR4271	C1QTNF5	miR665
DUSP22	miR1203, miR1275, miR1285, miR744, miR3162, miR4271	NYAP1	miR1285, miR4271
FMO4	miR2861	OPRM1	miR422a, miR3154, miR4271
ABO	miR92b	SOX15	miR146a
TTLL9	miR665	PRDM9	miR665

ZNF620	miR1285	OBSCN	miR658, miR3154
USF1	miR513a, miR4271	RREB1	miR665
RTN3	miR146a, miR708, miR4271	XPO5	miR146a, miR3154, miR3162
CPSF2	miR1285, miR146a, miR605, miR708, miR92b, miR4271	SYNGAP1	miR1275, miR3154, miR4271
FGD5	miR1275, miR513a	SDC1	miR3162, miR4274
APOL4	miR494	IL1R1	miR665,miR3162, miR4271
NOTCH2NL	miR3154, miR4271	MBD6	miR665, miR92b, miR3154, miR4271
RAB7A	miR3154, miR4271	RAX	miR744, miR92b
SORBS2	miR1275, miR1285, miR665	DDX10	miR3154, miR3162, miR4271
DCLRE1C	miR1285, miR422a	ADAM11	miR665, miR744, miR4271
CUL9	miR1285, miR146a, miR665	PHF8	miR1285, miR665, miR4271
DLL3	miR1285	LIMD1	miR1285, miR513a, miR3154
FZD9	miR1285	ZNF81	miR1275, miR1285, miR141, miR3162
GPX7	miR513a	PNMAL2	miR1275, miR1285, miR658, miR744, miR3162, miR3177
LOXL3	miR92b	AAK1	miR1275, miR1285, miR141, miR513a, miR665, miR3162, miR4271
MYNN	miR146a	NCR3	miR1275, miR4271
YAP1	miR1285, miR141, miR665, miR4271	ALG1	miR513a
DCBLD2	miR665	LIMS2	miR744, miR4271
LYPLA2	miR1285, miR513a, miR665	POLL	miR1285, miR141, miR3154
DCUN1D2	miR1285	SEC62	miR658, miR665, miR82b, miR4271
SLC5A6	miR1275, miR141, miR513a, miR658, miR4271	DGKZ	miR1203, miR665, miR4271
PHF21A	miR1285, miR665, miR4271		
